# Seasonal and spatial variability of zooplankton diversity in the Poyang Lake Basin using DNA metabarcoding

**DOI:** 10.1002/ece3.8972

**Published:** 2022-06-05

**Authors:** Xuemei Qiu, Xiongjun Liu, Quanfeng Lu, Jinping Chen, Tao Liang, Weikai Wang, Shan Ouyang, Chunhua Zhou, Xiaoping Wu

**Affiliations:** ^1^ 47861 School of Life Sciences Nanchang University Nanchang China; ^2^ School of Life Sciences Jiangxi Science and Technology Normal University Nanchang China; ^3^ 118348 Guangdong Provincial Key Laboratory of Conservation and Precision Utilization of Characteristic Agricultural Resources in Mountainous Areas School of Life Science Jiaying University Meizhou China

**Keywords:** biodiversity, conservation, DNA metabarcoding, freshwater ecosystems, zooplankton

## Abstract

Freshwater ecosystems face multiple threats to their stability globally. Poyang Lake is the largest lake in China, but its habitat has been seriously degraded because of human activities and natural factors (e.g. climate change), resulting in a decline in freshwater biodiversity. Zooplankton are useful indicators of environmental stressors because they are sensitive to external perturbations. DNA metabarcoding is an approach that has gained significant traction by aiding ecosystem conservation and management. Here, the seasonal and spatial variability in the zooplankton diversity were analyzed in the Poyang Lake Basin using DNA metabarcoding. The results showed that the community structure of zooplankton exhibited significant seasonal and spatial variability using DNA metabarcoding, where the community structure was correlated with turbidity, water temperature, pH, total phosphorus, and chlorophyll‐a. These results indicated habitat variations affected by human activities and seasonal change could be the main driving factors for the variations of zooplankton community. This study also provides an important reference for the management of aquatic ecosystem health and conservation of aquatic biodiversity.

## Graphical abstract

Freshwater ecosystems face multiple threats to their stability globally. Habitat degradation is one of the drivers of global change in freshwater ecosystems. Poyang Lake is the largest lake in China, but its habitat has been seriously degraded because of human activities and natural factors, resulting in a decline in freshwater biodiversity. Zooplankton are useful indicators of environmental stressors because they are sensitive to external perturbations. Here, the seasonal and spatial variability in the zooplankton diversity were analyzed in the Poyang Lake Basin using DNA metabarcoding. The results showed that the number of operational taxonomic units (OTUs) of zooplankton per sample ranged from 45 to 301. The dominant taxa of zooplankton were rotifers. The community structure of the zooplankton exhibited significant seasonal and spatial variability in the analysis through the non‐metric multidimensional scaling (NMDS) ordination plots and Bray‐Curtis resemblance matrix. The community composition of the zooplankton was correlated with turbidity, water temperature, pH, total phosphorus, and chlorophyll‐a using redundancy analysis (RDA). These results provides new insights on the seasonal and spatial dynamics of zooplankton communities in the Poyang Lake Basin, and provides an important reference for improves the management of aquatic ecosystem health and conservation of aquatic biodiversity.

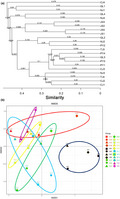

## 1 INTRODUCTION

Habitat degradation is one of the most important driving factors that pose serious threats to global biodiversity (Aguilar et al., 2008; Arroyo‐Rodrıguez et al., 2017; Laurance et al., 2002). For example, 52% of the biodiversity declined between 1970 and 2010, and this loss in the freshwater ecosystems was even greater than in the marine or terrestrial ecosystems (WWF, 2014). Additionally, for many communities, the response of other freshwater communities to environmental change is largely unknown (Celik et al., 2019; Gomes et al., 2019). Therefore, knowledge of accurate biodiversity estimates is important for effective conservation and management of natural resources (Dudgeon et al., 2006).

Zooplankton play an important role in the biogeochemical cycling of carbon (C) and nitrogen (N) and aid the stability of food webs in freshwater ecosystems (Walsh et al., 2018). Zooplankton are useful indicators of environmental stressors because they are sensitive to external perturbations such as climate change, habitat degradation, and organic pollution (Stefanni et al., 2018). Therefore, the biomass and species of zooplankton have been widely used in biological water monitoring (Stefanni et al., 2018). However, knowledge of the effect of environmental change on zooplankton communities is hindered by traditional taxonomy challenges (Djurhuus et al., 2018; Machida et al., 2009). Traditional species identification methods and morphology‐based individual counting methods are costly and time‐consuming, requiring trained personnel with expertise in identifying zooplankton, especially in large‐scale environmental investigations and monitoring programs (Ren et al., 2019). Traditional biomonitoring methods apply only to species that are easily observed (Walczyńska et al., 2019). For some taxonomic groups, it is difficult or almost impossible to identify the species through morphological methods (Choquet et al., 2018). Therefore, it has become evident that morphological methods do not meet the increasing demand for biodiversity monitoring used in conservation and management decisions.

DNA metabarcoding is an approach that has gained significant traction by aiding ecosystem conservation and management (Goldberg et al., 2016; Taberlet et al., 2012; Thomsen & Willerslev, 2015), and has the potential to greatly reduce cost and time (Thomsen & Willerslev, 2015). Recently, DNA metabarcoding has been widely used for the detection of many taxa in freshwater ecosystems (Deiner et al., 2015; Hänfling et al., 2016; Lopes et al., 2017; Thomsen et al., 2012; Valentini et al., 2016). To date, compared to traditional monitoring, DNA metabarcoding research has demonstrated higher detection capability and cost‐effectiveness (Sigsgaard et al., 2015), and it has provided the power to detect invasive and rare species (Dejean et al., 2012; Elbrecht et al., 2018; Piaggio et al., 2014; Sigsgaard et al., 2016). Therefore, DNA metabarcoding may solve the traditional taxonomy challenges of zooplankton and reduce cost and time in large‐scale environmental investigations and monitoring programs (Iacchei et al., 2017; Pawlowski et al., 2020; Thomsen & Willerslev, 2015), yet the limitations of the approaches to acquiring data and the existing geographical bias need to be considered (Belle et al., 2019; Bucklin et al., 2016; Rey et al., 2020; Stoeckle et al., 2016).

Poyang Lake, the largest freshwater lake in China, is one of two lakes connected to the Yangtze River, and is a biodiversity hotspot of freshwater species (Huang et al., 2013; Jin et al., 2012). It plays an important role in maintaining and supplementing freshwater biodiversity for the Yangtze River because of extremely abundant aquatic organisms (Jin et al., 2012; Li et al., 2019; Liu, Liu, et al., 2019; Liu, Qin, et al., 2019) Poyang Lake is also a dynamic wetland system, forming a large lake covering more than 3000 km^2^ with a high water level in the rainy season of summer and covering <1000 km^2^ with a low water level in the dry season of winter (Jin et al., 2012; Li et al., 2019). However, in recent years, this lake has been confronted with shrinkage and environmental problems due to anthropogenic habitat disturbances, resulting in the decline of aquatic biodiversity (Huang et al., 2013; Jin et al., 2012; Li et al., 2019). Poyang Lake has suffered from water quality degradation with significantly increasing eutrophication (Liao et al., 2017; Liu et al., 2020). The lake area has declined from 5200 km^2^ in 1949 to 3287 km^2^ in the 21st century (Han et al., 2014; Li et al., 2019). Due to the Three Gorges Dam reducing discharge, seasonal water shortages also occurred frequently (Lai et al., 2014), and affected the survival of freshwater species (Min & Zhan, 2012). In recent years, the fluctuations in water level changed dramatically and occurred an early seasonal drying in lake areas (Feng et al., 2016; Mei et al., 2016). To understand these degradation issues, it is imperative to assess the status of the ecosystem. Previous research points to the seasonal and spatial variability in zooplankton diversity using traditional monitoring methods in Poyang Lake Basin (Chen et al., 2020; Lu et al., 2021; Lv, 2019), but no study used DNA metabarcoding to analyze the seasonal and spatial variability in zooplankton diversity. Here, we aimed to analyze the seasonal and spatial variability in zooplankton diversity using DNA metabarcoding and to explore the correlation between environmental parameters and zooplankton community composition. We test whether it had significantly seasonal and spatial variability in zooplankton diversity using DNA metabarcoding, and whether it had differed from those traditional monitoring methods? This study provides an important reference for the management of aquatic ecosystem health and conservation of aquatic biodiversity.

## 2 MATERIALS AND METHODS

### 2.1 Study area

**FIGURE 1:**
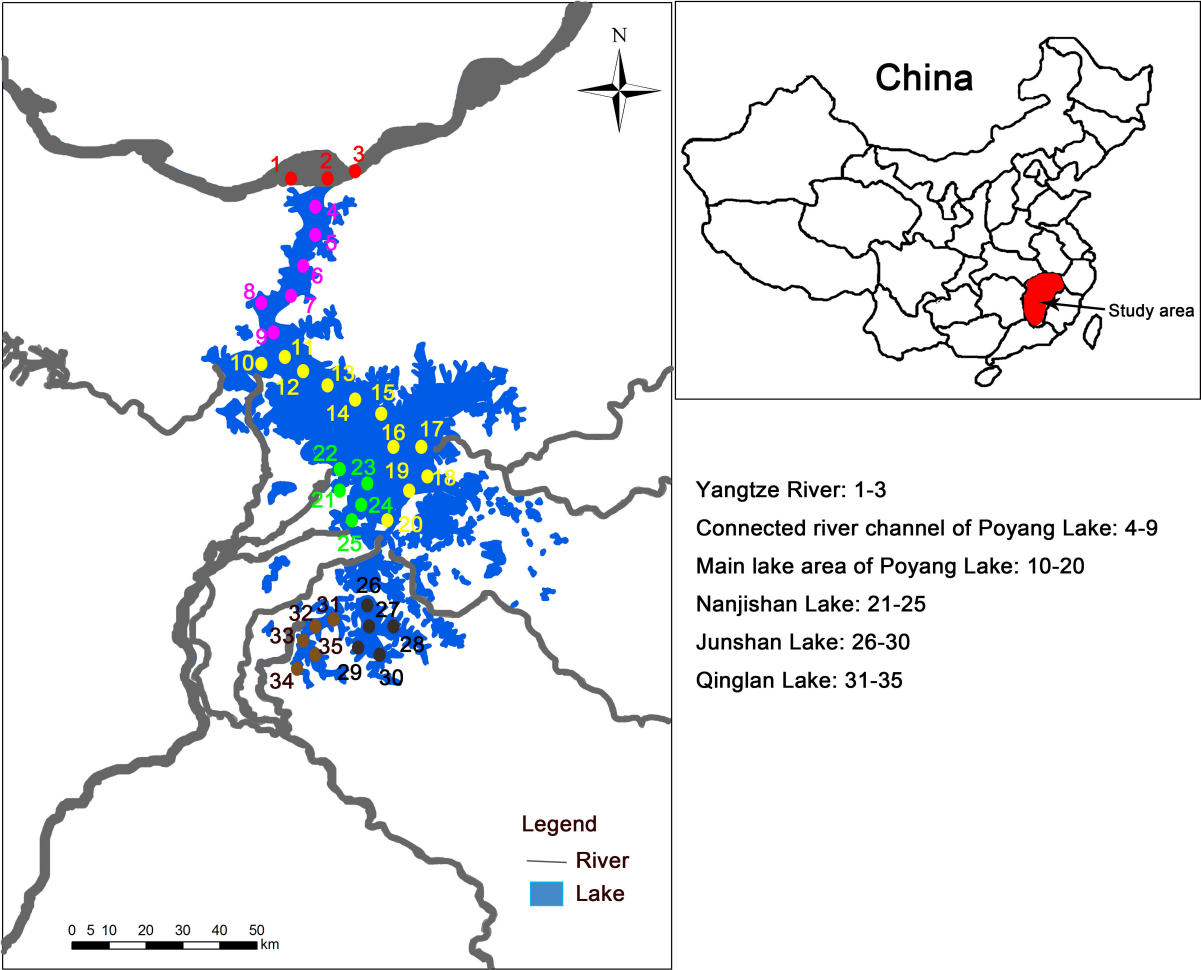
Location of the sampling sections for zooplankton in the Poyang Lake Basin

Poyang Lake is the largest freshwater lake in China and is connected to the middle reaches of the Yangtze River (Figure 1; Jin et al., 2012). The Poyang Lake Basin has a total area of 16.2 × 10^4^ km^2^, an average annual precipitation of 1350–2150 mm, and a surface runoff of 1457 × 10^8^ m^3^. In this study, we considered habitat variation and anthropogenic activities for the selection of sampling areas in the Poyang Lake Basin. We established six sampling sections in the Poyang Lake Basin in April (spring), July (summer), October (autumn) 2019, and January (winter) 2020: the Yangtze River (CJ; 1–3); the connected river channel of Poyang Lake (TJ; 4–9); the main lake area of Poyang Lake (PY; 10–20); Nanjishan area of Poyang Lake (NJ; 21–25); Junshan Lake (JS; 26–30) and Qinglan Lake (QL; 31–35; for anthropogenic activities and substrates details see Table 1). Due to rapid water flow in the connected river channel of Poyang Lake, we did not collect the water samples of zooplankton in the spring and summer of 2019.

**TABLE 1:** Mean physicochemical parameters of water quality from the six sampling sections in the Poyang Lake Basin (mean ± SD)

Parameters	Yangtze River	Connected river channel of Poyang Lake	Main lake area of Poyang Lake	Junshan Lake	Qinglan Lake	Nanjishan area of Poyang Lake
	Mean ± SD	Mean ± SD	Mean ± SD	Mean ± SD	Mean ± SD	Mean ± SD
WD (m)	13.83 ± 4.06	8.96 ± 1.31	5.9 ± 1.01	5.03 ± 0.47	3.45 ± 1.22	1.67 ± 0.45
V (m/s)	0.38 ± 0.09	0.31 ± 0.04	0.21 ± 0.04	0.15 ± 0.03	0.17 ± 0.04	0.10 ± 0.02
Turb (NTU^+^)	13.5 ± 4.39	26.5 ± 6.15	13.16 ± 0.75	6.55 ± 3.68	30.67 ± 18.16	73.72 ± 21.83
T (℃)	19.85 ± 2.96	19.72 ± 3.63	19.63 ± 4.08	20.29 ± 4.33	21.43 ± 3.94	19.31 ± 3.95
Sal (mg/L)	0.13 ± 0.03	0.05 ± 0.01	0.04 ± 0.01	0.08 ± 0.03	0.06 ± 0.01	0.05 ± 0.01
DO (mg/L)	8.75 ± 0.11	8.71 ± 0.05	8.16 ± 0.25	8.46 ± 0.31	7.66 ± 0.38	7.56 ± 0.23
Chl‐a (μg/L)	5.11 ± 1.56	17.08 ± 1.99	16.69 ± 4.08	10.28 ± 3.72	37.98 ± 11.88	25.28 ± 4.97
pH	6.8 ± 0.31	6.67 ± 0.11	6.83 ± 0.12	7.32 ± 0.41	7.09 ± 0.27	7.21 ± 0.25
TN (mg/L)	1.92 ± 0.03	1.75 ± 0.12	1.65 ± 0.17	1.98 ± 0.48	0.92 ± 0.12	1.74 ± 0.2
TP (mg/L)	0.15 ± 0.03	0.16 ± 0.02	0.16 ± 0.01	0.22 ± 0.05	0.11 ± 0.01	0.18 ± 0.01
Anthropogenic activities	Sand mining, industrial pollution, and urban development	Sand mining and urban development	Sand mining and overfishing	Aquaculture	Aquaculture and overfishing	Aquaculture
Substrates	Sand	Hard mud, sand, and silt	Hard mud, sand, and silt	Silt	Silt and sand	Silt

Abbreviations: Chl‐a, chlorophyll‐a; DO, dissolved oxygen; Sal, salinity; T, water temperature; TN, total nitrogen; TP, total phosphorus; Turb, turbidity; V, water velocity; WD, water depth.

### 2.2 Sample collection

At per sampling site, 20 L quantitative water samples of zooplankton were collected using 64 µm mesh size net from a bottom depth, just above the sediment, and at the surface (i.e., 0.5 m) were passed in the field. In the laboratory, three quantitative samples of zooplankton from the Yangtze River were further mixed and filtered through 5‐μm microporous filter paper (Millipore) in April (spring), July (summer), October (autumn) 2019, and January (winter) 2020, respectively. Filter membranes were then placed in a 5‐ml centrifuge tube. Finally, a total of four samples from the Yangtze River were used for DNA metabarcoding analysis and stored at −20°C until extraction of DNA (Table S1). Similarly, for other sampling sections, we used the same methods to obtain samples used for DNA metabarcoding analysis (Table S1). Therefore, a total of 22 samples from the Poyang Lake Basin were used for DNA metabarcoding analysis.

### 2.3 DNA extraction, PCR amplification, and high‐throughput sequencing

Genomic DNA from the 22 samples was extracted using the TIANamp Marine Animals DNA Kit (TianGen). The concentration and quality of DNA were estimated using a Nanodrop 2000 spectrophotometer (Thermo Scientific) and agarose gel electrophoresis.

DNA metabarcoding of mitochondrial COI 313 bp region was used to analyze the seasonal and spatial variability in zooplankton diversity. PCR amplification of the cytochrome c oxidase subunit I (COI) genes was performed using the forward primer sequence mlCOIintF (5′‐ GGWACWGGWTGAACWGTWTAYCCYCC ‐3′), and the reverse primer sequence HCO700DY2 (5′‐ TAAACTTCAGGGTGACCAAAAAATCA ‐3′) (Leray et al., 2013). Sample‐specific 7‐bp barcodes were incorporated into the primers for multiplex sequencing at the library preparation part. The PCR reaction was carried out in a 25 μl volume containing 5 µl of 5 × buffer, 14.75 µl of ddH_2_O, 1 µl of 10 µM forward primer, 1 µl of 10 µM reverse primer, 2 µl of 2.5 mM deoxyribonucleotide triphosphates, 0.25 µl fast pfu DNA polymerase, and 1 µl of genomic DNA. Triplicate PCR reactions were performed for each sample to minimize the potential bias of the PCR. Sterile water was used as a negative control in the study and the strategies were employed in sterile operating table of the laboratory to prevent DNA contamination. The PCR amplifications were conducted for an initial denaturation at 98°C for 5 min, followed by 27 cycles of 98°C for 30 s, annealing temperature of 50°C for 30 s, 72°C for 45 s, and a final extension at 72°C for 5 min. PCR products were detected on a 2% agarose gel, and fragments from the gel were purified with Agencourt AMPure Beads (Beckman Coulter). After purification on the gel, products of PCR were quantified using the PicoGreen dsDNA Assay Kit (Invitrogen).

The PCR amplification products were sequenced using the Illumina MiSeq platform from the Shanghai Personal Biotechnology Co., Ltd (Degnan & Ochman, 2012). Libraries were prepared using Illumina's TruSeq Nano DNA LT Library Prep Kit. The PCR amplification products were pooled to form a library for sequencing. Equimolar PCR products from each sample were used to ensure an equal contribution of each community in the final sequencing library. An Illumina MiSeq platform was used based on a paired‐end 300 bp sequence read run after library preparation.

### 2.4 Bioinformatics

The paired‐end sequences were assembled using the FLASH software (http://ccb.jhu.edu/software/FLASH/; Magoc & Salzberg, 2011). Raw FASTQ files were demultiplexed and quality filtered using QIIME 2 (Bolyen et al., 2019), and reads of low quality (mean quality <20, scanning window = 50; contained ambiguous ‘N’; sequence length: ≥150 bp) were discarded. Mothur software (Edgar, 2010; Quast et al., 2013) was used to cluster operational taxonomic units (OTUs) of zooplankton with a 97% similarity cutoff, and QIIME2 (Bolyen et al., 2019) was used to generate rarefaction curves. According to a reference database (NCBI nucleotide database in Genbank; Greengenes database (Release 13.8, http://greengenes.secondgenome.com/), DeSantis et al., 2006; RDP (Ribosomal Database Project) database (Release 11.1, http://rdp.cme.msu.edu/), Cole et al., 2009; Silva database (Release132, http://www.arb‐silva.de), Quast et al., 2013; UNITE database (Release 7.0, https://unite.ut.ee/), Koljalg et al., 2013), we used the Statistical Assignment Package (SAP version 1.3.2; Munch et al., 2008) to assign the representative sequence from each zooplankton OTU to a specific taxonomic group. SAP was used to retrieve homologs in each query sequence. The phylogenetic trees, taxonomy compositions, and abundances were visualized using MEGAN (Huson et al., 2011). The posterior probability was calculated for the query sequence to belong to a taxonomic group at phylum, class, order, family, genus, and species levels of zooplankton, respectively. The assignments at a significance level of 60% (posterior probability) were accepted, and SAP to retrieve 100 homologs at >80% sequence similarity was allowed. Alpha diversity indices, such as Chao1 richness estimator, ACE metric (Abundance‐based Coverage Estimator), Shannon diversity index, and Simpson index, were calculated using the combined OTU‐tables of the same species table of zooplankton in Mothur software (Edgar, 2010; Quast et al., 2013). The non‐metric multidimensional scaling (NMDS) ordination plots were used to assess the variation in the zooplankton community among sampling sections. The Bray–Curtis resemblance matrix of the zooplankton community from sampling sections was generated and represented by the NMDS ordination plots. The NMDS ordination plots and Bray–Curtis resemblance matrix were generated using R version 2.13.1 (R Development Core Team, 2011) and the VEGAN package (Oksanen et al., 2015). One‐way analysis of variance (ANOVA) was used to detect differences in the OTUs, alpha diversity indices, and environmental factors between each section and each season. We used post hoc tests to make further comparisons. We used Tukey's honestly significant difference tests for these comparisons, but in cases of persistent heteroscedasticity (i.e., when Levene's test was significant) we used Games–Howell tests because they do not assume equal variances between groups. SPSS version 22.0 was used to perform the ANOVA tests.

### 2.5 Measurement of physicochemical parameters

We used four water quality variables to analyze changes in the environmental factors in the Poyang Lake Basin in April (spring), July (summer), October (autumn) 2019, and January (winter) 2020. We used a YSI 650MDS (YSI) multiparameter meter to measure the water temperature (°C), dissolved oxygen (mg/L), pH, salinity (mg/L), and turbidity (NTU^+^). Chlorophyll‐a concentration (mg/L) was measured using a chlorophyll meter (PCH‐800). A velocity meter (FP111, Global Water, 0.1 m/s accuracy) was used to measure the water velocity, and a digital sonar system (H22px handheld sonar system) was used to measure the water depth (m). In addition, concentrated sulfuric acid (H_2_SO_4_) was used to preserve the collected water samples. These collected water samples for nutrient analysis were then refrigerated and transported to the Nanchang University laboratory. The total nitrogen (TN; mg/L) and total phosphorus (TP; mg/L) content were analyzed using ultraviolet spectrophotometry (Huang et al., 1999; Wei et al., 1989).

### 2.6 Correlation between environmental factors and zooplankton community structure

We performed a detrended correspondence analysis for the composition of zooplankton community to determine whether linear or unimodal ordination (Lep & Smilauer, 2003). To evaluate the correlation between environmental factors and community composition of the zooplankton, a redundancy analysis (RDA) with 499 Monte Carlo permutations was performed using CANOCO version 4.5 (ter Braak & Verdonschot, 1995; Lep & Smilauer, 2003). All environmental factors and community composition of zooplankton were log_10_(*X* + 1) transformed to meet the assumptions of multivariate normality and to moderate the influence of extreme data (Borcard et al., 2011).

## 3 RESULTS

### 3.1 The OTUs of zooplankton

A total of 1,197,035 raw sequences were generated from 22 samples (NCBI SRA Accession no. PRJNA661399). A total of 338,947 sequences (28.3%) were obtained after quality filtering and 240,053 sequences belonged to the zooplankton. The sequence number of each OTU sample was distributed in the 97% sequence similarity threshold based on Chao1 and Shannon rarefaction curves (Figure S1). The number of total OTUs per sample ranged from 72 to 355, and the number of zooplankton OTUs per sample ranged from 45 to 301 (Table 2). Significant differences were detected in the number of zooplankton OTUs in each season (ANOVA, *p* < .05). The number of zooplankton OTUs in spring and summer was greater than that in autumn and winter (Table 2; Table S2; Figure S2). In addition, we also found significant differences in the number of zooplankton OTUs among each sampling area (ANOVA, *p* < .05). The number of zooplankton OTUs in the main lake areas of Poyang Lake and Nanjishan area of Poyang Lake were greater than those in the other sampling areas (Table 2; Table S2; Figure S2).

**TABLE 2:** Seasonal and spatial variability of total and zooplankton sequences and OTUs, and alpha diversity indices of zooplankton in the Poyang Lake Basin

Sampling areas	Time	Code	Alpha diversity indices of zooplankton				Total sequences	Total OTUs	Zooplankton sequences	Zooplankton OTUs
			Simpson	Chao1	ACE	Shannon				
Yangtze River	Spring	CJ1	0.82	143.56	144.37	3.65	16,565	171	5927	108
	Summer	CJ2	0.77	104.88	108.52	3.31	13,298	107	5694	62
	Autumn	CJ3	0.84	66.00	68.33	3.77	13,420	72	12,414	58
	Winter	CJ4	0.63	63.40	67.03	2.34	16,348	76	13,671	45
	Mean		0.76	94.46	97.06	3.27	14,908	107	9427	68
Main lake area of Poyang Lake	Spring	PY1	0.88	231.57	243.69	4.46	13,076	255	9451	198
	Summer	PY2	0.91	231.16	249.31	4.49	17,320	300	14,724	262
	Autumn	PY3	0.78	131.97	134.35	3.29	16,649	166	10,546	133
	Winter	PY4	0.81	128.61	134.77	3.62	15,320	146	10,140	133
	Mean		0.84	180.83	190.53	3.97	15,591	217	11,215	181
Nanjishan area of Poyang Lake	Spring	NJ1	0.92	148.45	152.68	4.69	17,046	181	16,535	160
	Summer	NJ2	0.91	277.11	288.74	4.89	17,234	211	16,892	193
	Autumn	NJ3	0.89	178.13	179.02	4.48	17,140	238	11,316	194
	Winter	NJ4	0.83	91.00	93.14	3.78	16,840	101	16,755	90
	Mean		0.89	173.67	178.40	4.46	17,065	183	15,375	159
Junshan Lake	Spring	JS1	0.22	65.00	74.27	0.96	17,374	90	1702	63
	Summer	JS2	0.85	167.69	175.65	4.03	17,042	355	14,050	301
	Autumn	JS3	0.86	95.87	100.46	3.54	17,309	130	11,417	111
	Winter	JS4	0.65	87.67	91.86	2.63	17,098	117	7304	103
	Mean		0.65	104.06	110.56	2.79	17,206	173	8618	145
Qinglan Lake	Spring	QL1	0.58	120.88	122.94	2.87	15,716	148	14,180	112
	Summer	QL2	0.92	256.52	271.14	4.91	11,893	271	8710	221
	Autumn	QL3	0.70	139.05	139.37	3.16	10,973	142	10,170	123
	Winter	QL4	0.93	134.08	134.58	4.94	7814	152	7317	138
	Mean		0.78	162.63	167.01	3.97	11,599	178	10,094	149
Connected river channel of Poyang Lake	Autumn	TJ3	0.77	125.69	131.55	3.24	17,091	188	16,252	164
	Winter	TJ4	0.54	93.88	95.08	2.24	16,381	118	4886	86
	Mean		0.66	109.79	113.32	2.74	16,736	153	10,569	125

### 3.2 Seasonal and spatial variability in the diversity of zooplankton

**FIGURE 2:**
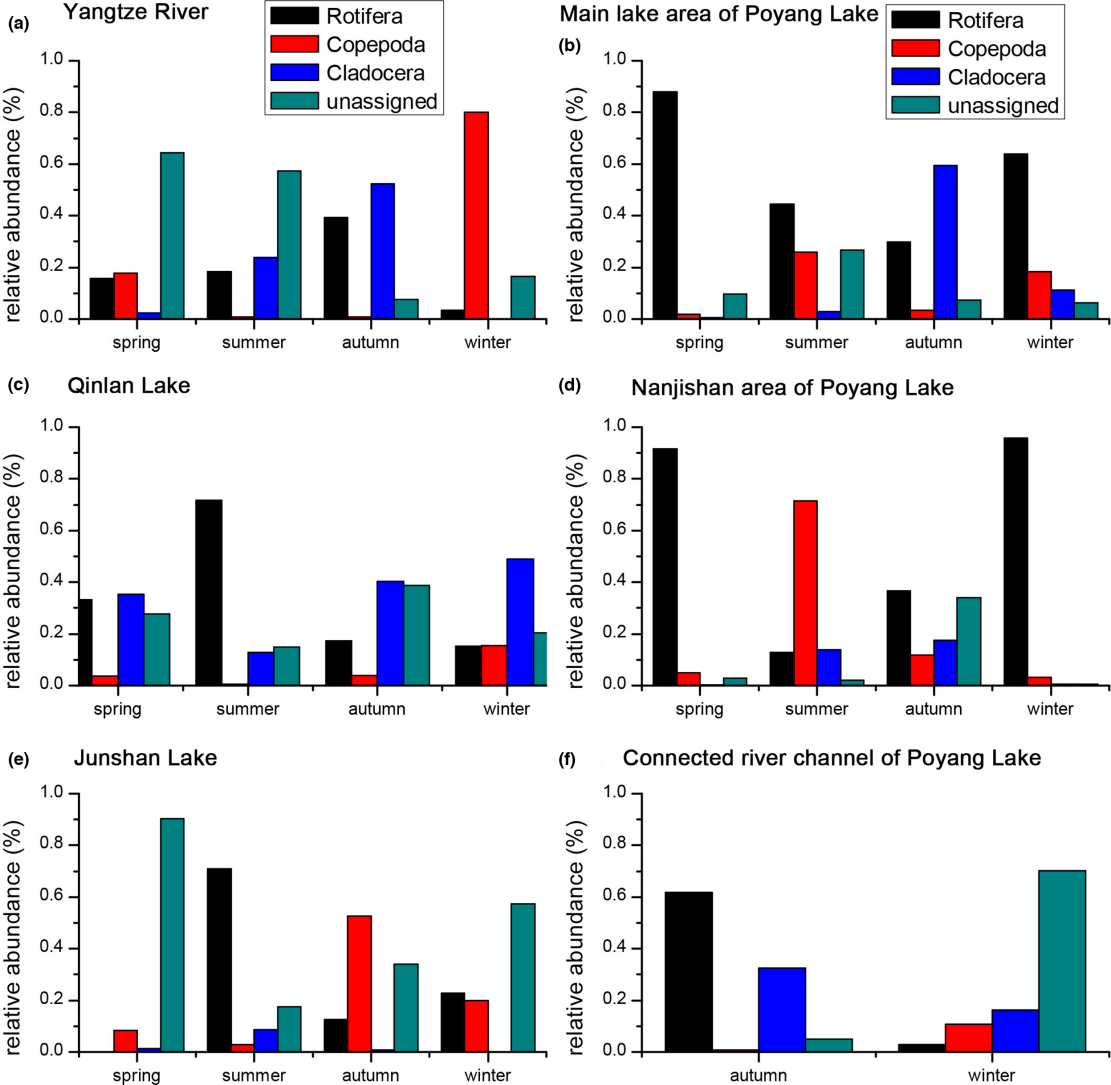
Seasonal changes in the relative abundance of zooplankton found in the Yangtze River (a), main lake area of Poyang Lake (b), Qinlan Lake (c), Nanjishan area of Poyang Lake (d), Junshan Lake (e), and connected river channel of Poyang Lake (f)

The combined the OTUs of the same zooplankton species were categorized into 92 species, 45 genera, 26 families, eight orders, four classes, and two phyla in the Poyang Lake Basin (Table S3). Of the total zooplankton species detected 52.2% were rotifera, 29.3% were copepods, and 18.5% were cladocerans. Significant differences were detected in the relative abundance of zooplankton in each season (ANOVA, *p* < .05). The relative abundance of rotifera in spring and summer was greater than that in autumn and winter (Figure 2; Figure S3). The relative abundance of copepods in winter and cladocerans in autumn was greater than that in other seasons (Figure 2; Figure S3). In addition, we also found significant differences in the relative abundance of zooplankton in each sampling area (ANOVA, *p* < .05). The relative abundance of rotifera in the Qinlan Lake and Nanjishan area of Poyang Lake was greater than that in the other sampling areas (Figure 2; Figure S3). The relative abundance of copepods in the Junshan Lake was greater than that in the other sampling areas (Figure 2; Figure S3). The relative abundance of cladocerans in the main lake area of Poyang Lake was greater than that in the other sampling areas (Figure 2; Figure S3).

Significant differences were detected in the diversity of zooplankton among the different seasons (ANOVA, *p* < .05). The diversity of zooplankton in the summer (Simpson = 0.87; Chao1 = 207.5; ACE = 218.7; Shannon = 4.3) was greater than those in the other seasons (Table 2). We also found significant differences in the diversity of zooplankton among the sampling areas (ANOVA, *p* < .05). The diversity of zooplankton in the main lake areas of Poyang Lake (Simpson = 0.84; Chao1 = 180.83; ACE = 190.53; Shannon = 3.97) and Nanjishan area of Poyang Lake (Simpson = 0.89; Chao1 = 173.67; ACE = 178.40; Shannon = 4.46) were greater than those in the other sampling areas (Table 2).

### 3.3 Community structure of zooplankton

**FIGURE 3:**
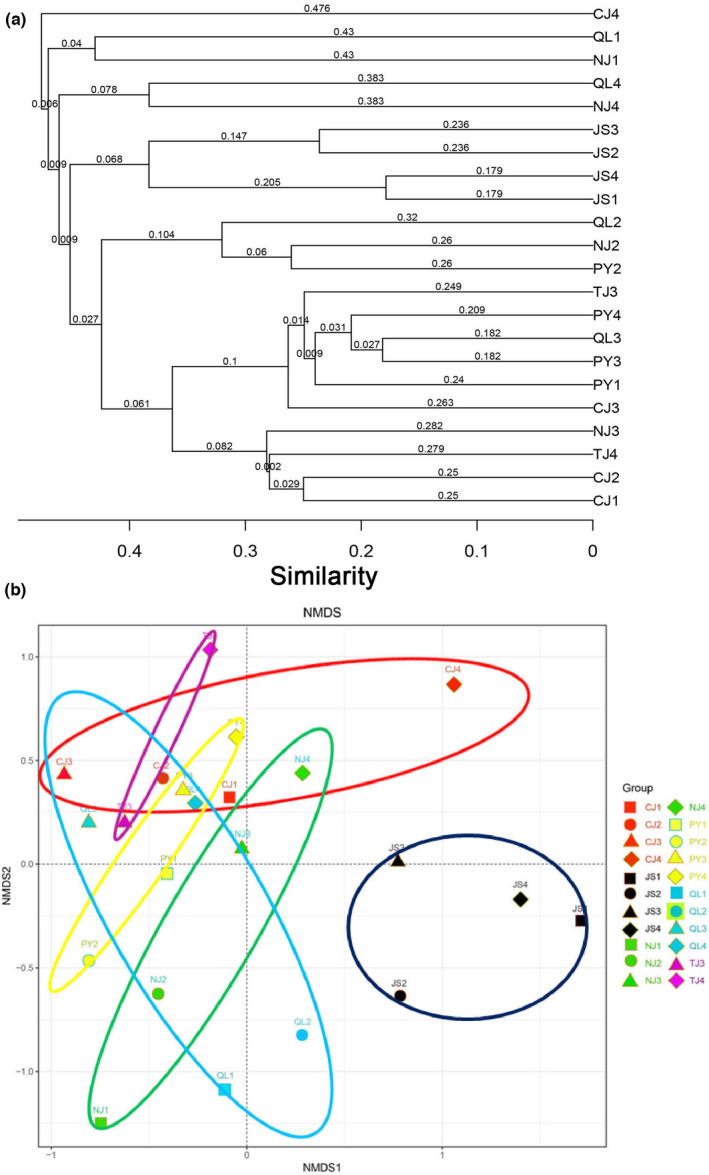
The Bray–Curtis resemblance matrix (a) and the non‐metric multidimensional scaling (NMDS) ordination (b) in the community structure of zooplankton in the Poyang Lake Basin. Sampling section codes are as in Table 2

The Bray–Curtis resemblance matrix showed that the community structure of zooplankton in spring was divided into three areas: the first area included the Nanjishan area of Poyang Lake and Qinlan Lake, the second area included the Yangtze River and the main lake area of Poyang Lake, and the third area included the Junshan Lake (Figure 3). The community structure of zooplankton in summer was divided into three areas, in which the first area included the main lake area of Poyang Lake, Nanjishan area of Poyang Lake, and Qinlan Lake, the second area included the Yangtze River, and the third area included the Junshan Lake (Figure 3). The community structure of zooplankton in autumn was divided into four areas, in which the first area included the Qinlan Lake and the main lake area of Poyang Lake, the second area included the Junshan Lake, the third area included the Nanjishan area of Poyang Lake and the Yangtze River, and the fourth area included the connected‐river channel of Poyang Lake (Figure 3). The community structure of zooplankton in winter was divided into five areas, in which the first area included the Qinlan Lake and Nanjishan area of Poyang Lake, the second area included the Yangtze River, the third area included the connected‐river channel of Poyang Lake, the fourth area included the main lake area of Poyang Lake, and the fifth area included the Junshan Lake (Figure 3). The results of the NMDS plot were coincident with the Bray–Curtis resemblance matrix, indicating that the results were reliable (stress = 0.11; Figure 3).

### 3.4 Correlation between the community composition of zooplankton and environmental factors

**FIGURE 4:**
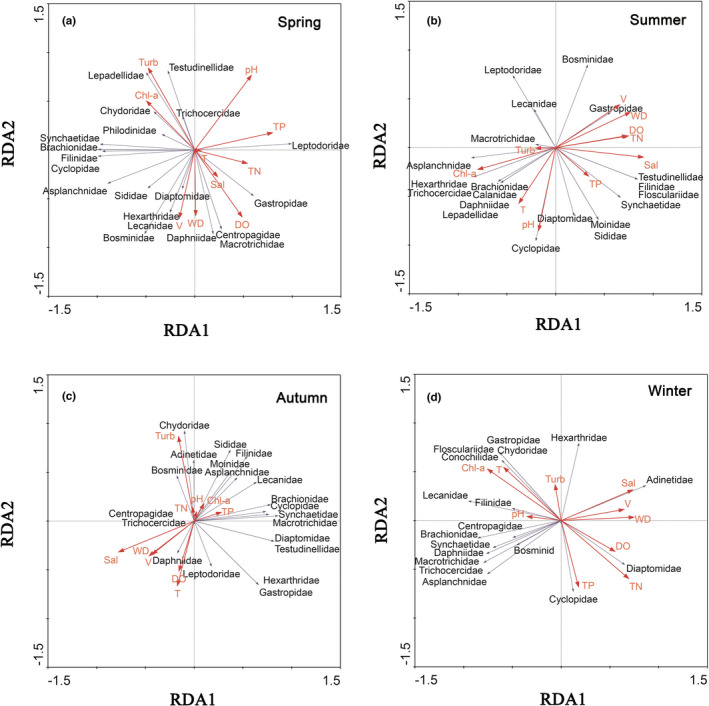
Analysis of correlation between the environmental factors and zooplankton community in spring (a), summer (b), autumn (c), and winter (d). Chl‐a, chlorophyll‐a; DO, dissolved oxygen; Sal, salinity; T, water temperature; TN, total nitrogen; TP, total phosphorus; Turb, turbidity; V, water velocity; WD, water depth

Significant differences were detected in the water depth, turbidity, dissolved oxygen, chlorophyll‐a, and salinity among the sampling areas (ANOVA, *p* < .05; Table 1). Additionally, significant differences were found in the water depth, temperature, total nitrogen, and velocity between the seasons (ANOVA, *p* < .05; Table 1). Redundancy analysis showed that Leptodoridae, Gastropidae, Centropagidae, Macrotrichidae, Daphniidae, Bosminidae, Lecanidae, Hexarthridae, and Diaptomidae in spring were correlated with water depth, velocity, salinity, dissolved oxygen, and total nitrogen (Figure 4a). Sididae, Asplanchnidae, Cyclopidae, Filinidae, Brachionidae, Synchaetidae, Philodinidae, and Chydoridae in spring were correlated with water velocity and chlorophyll‐a (Figure 4a). Lepadellidae, Trichocercidae, and Testudinellidae in spring were correlated with turbidity, pH, and chlorophyll‐a (Figure 4a). Diaptomidae, Sididae, Moinidae, Synchaetidae, Flosculariidae, Filinidae, and Testudinellidae in summer were correlated with dissolved oxygen, salinity, total phosphorus, and total nitrogen (Figure 4b). Asplanchnidae, Hexarthridae, Trichocercidae, Brachionidae, Calanidae, Daphniidae, Lepadellidae, and Cyclopidae in summer were correlated with turbidity, water temperature, pH, and chlorophyll‐a (Figure 4b). Leptodoridae, Lecanidae, and Macrotrichidae in summer were correlated with turbidity and chlorophyll‐a (Figure 4b). Gastropidae and Bosminidae in summer were correlated with dissolved oxygen, water depth, water velocity, and total nitrogen (Figure 4b). Macrotrichidae, Synchaetidae, Cyclopidae, Brachionidae, Lecanidae, Asplanchnidae, Moinidae, Chydoridae, Sididae, Bosminidae, Adinetidae, and Filinidae in autumn were correlated with turbidity, total phosphorus, total nitrogen, pH, and chlorophyll‐a (Figure 4c). Diaptomidae, Testudinellidae, Hexarthridae, Leptodoridae, and Gastropidae in autumn were correlated with water temperature, total phosphorus, and dissolved oxygen (Figure 4c). Diaptomidae, Testudinellidae, Hexarthridae, Leptodoridae, and Gastropidae in autumn were correlated with water temperature, total phosphorus, and dissolved oxygen (Figure 4c). Centropagidae, Daphniidae, and Trichocercidae in autumn were correlated with salinity, water depth, water velocity, water temperature, and dissolved oxygen (Figure 4c). Leptodoridae, Gastropidae, Centropagidae, Macrotrichidae, Daphniidae, Bosminidae, Lecanidae, Hexarthridae Sididae, Asplanchnidae, Filinidae, Brachionidae, Synchaetidae, Philodinidae, Chydoridae, Lepadellidae, Trichocercidae, and Testudinellidae in winter were correlated with water temperature, pH, and chlorophyll‐a (Figure 4d). Cyclopidae and Diaptomidae in winter were correlated with total phosphorus, water depth, water velocity, total nitrogen, and dissolved oxygen (Figure 4d). Hexarthridae and Adinetidae in winter were correlated with water depth, water velocity, salinity, and turbidity (Figure 4d).

## 4 DISCUSSION

### 4.1 Seasonal and spatial variability of zooplankton diversity

Knowledge of accurate biodiversity estimates is important for effective conservation and management of natural resources (Dudgeon et al., 2006). Improved biodiversity monitoring programs are important for maintaining the integrity of freshwater ecosystems (Dudgeon et al., 2006). DNA metabarcoding has been widely used for the detection of many taxa in freshwater ecosystems (Lopes et al., 2017; Valentini et al., 2016). Understanding the potential of DNA metabarcoding to identify aquatic biodiversity and the distribution dynamics in freshwater ecosystems is important for improving biodiversity monitoring (Thomsen & Willerslev, 2015). In this study, to determine the seasonal and spatial zooplankton variations and association of water quality, the diversity of zooplankton was analyzed using DNA metabarcoding in the Poyang Lake Basin. The results showed that the combined OTU‐table of the same zooplankton species from the Poyang Lake Basin was categorized into 92 species, 45 genera, 26 families, eight orders, four classes, and two phyla using DNA metabarcoding, which was similar to a recent study using traditional biomonitoring methods (Chen et al., 2020; Lu et al., 2021; Lv, 2019). In addition, rotifers constitute the most diverse group within the zooplankton community using traditional biomonitoring methods (Chen et al., 2020; Hu et al., 2019; Lu et al., 2021; Lv, 2019; Qin et al., 2020), and DNA metabarcoding in this study also revealed rotifers as the most diverse group.

Significant differences in the diversity of zooplankton were found among the different seasons. The diversity of zooplankton in spring and summer was greater than those in autumn and winter, which was similar to those in studies based on traditional biomonitoring methods (Chen et al., 2020; Lu et al., 2021; Lv, 2019). Such temporal distribution patterns of zooplankton in the Poyang Lake Basin have also been reported by previous microscopy‐based studies (Chen et al., 2020; Lu et al., 2021; Lv, 2019). The temporal distribution in the relative abundance of zooplankton major groups was also consistent with the plankton ecology group model (PEG model emphasized the role of physical factors, grazing and nutrient limitation for phytoplankton, and the role of food limitation and fish predation for zooplankton; Sommer et al., 2012). It may be related to the seasonality in most subtropical lakes and rivers (Scarabotti et al., 2017; Srifa et al., 2016). The synergistic coupling between the change in season and water level led to seasonal variation in the zooplankton community in the Poyang Lake Basin.

Significant differences in the diversity of zooplankton were found among the sampling areas. The diversity of the zooplankton in the main lake area of Poyang Lake and the Nanjishan area of Poyang Lake (southern district (area) in Poyang Lake) were greater than those in the other sampling areas. Spatial changes in the zooplankton in our study were similar to those in studies based on traditional biomonitoring methods (Chen et al., 2020; Lu et al., 2021; Lv, 2019). Some studies have shown that habitat variability of the Poyang Lake Basin could affect the community structure of zooplankton based on traditional biomonitoring methods (Lu et al., 2021; Lv, 2019; Qin et al., 2020). Indeed, the habitat diversity of the lake area is higher than that of the other sampling areas. The lake area has abundant nutrients and a stable water body, which provides a good habitat for the growth of zooplankton (Liu et al., 2020). The relatively rapid water flow in the connected river channel of Poyang Lake and the Yangtze River is not conducive to the growth and survival of zooplankton (Li et al., 2019; Liu et al., 2020). Uncovering the environmental factors affecting the observed deterministic community dynamics of zooplankton is a key challenge. In this study, the community composition of zooplankton was correlated with turbidity, water temperature, pH, total phosphorus, and chlorophyll‐a, which was similar to studies based on traditional biomonitoring methods (Lu et al., 2021; Lv, 2019; Qin et al., 2020). Indeed, some studies have shown that environmental factors affected the community composition of zooplankton (Hu et al., 2014, 2019; Hussain et al., 2016; Trevisan & Forsberg, 2007). Water temperature is an important environmental factor that affects the composition of zooplankton community (Kagalou et al., 2010). For example, water temperature could affect the growth and reproduction of zooplankton (Hu et al., 2008, 2019). Hu et al. (2019) found that pH had a significant effect on the seasonal variation of the zooplankton community. This study also showed that pH negatively affected the community composition of zooplankton. Total phosphorus was strongly correlated with the biomass of algae, resulting in an increase in zooplankton production (Qin et al., 2020; Trevisan & Forsberg, 2007). Chlorophyll‐a and total phosphorus in spring and summer were the main environmental factors affecting the community composition of zooplankton in this study.

### 4.2 Effect of human activity on the seasonal and spatial variability of zooplankton diversity

The Poyang Lake Basin is one of the most human disturbance basins in China, and biodiversity conservation faces great challenges (Li et al., 2019; Liu, Liu, et al., 2019; Liu, Qin, et al., 2019; Zhang et al., 2020). Human activities have affected the Poyang Lake Basin's freshwater organisms and their habitats with continual socioeconomic development (Zhang et al., 2020). The degraded habitat in Poyang Lake Basin has seriously affected freshwater biodiversity (Li et al., 2019). The degradation process is driven by human activities, such as sand mining, dam construction, water pollution, and overfishing in the basin (Li et al., 2019; Liu, Liu, et al., 2019; Liu, Qin, et al., 2019). For example, the increasing concentrations of nutrients and heavy metals have resulted in water quality deterioration, which indirectly affected zooplankton diversity (Liu et al., 2020; Lu et al., 2021). Sand mining has changed the physicochemical factors of water, affecting the zooplankton community (Johnson et al., 2012; Narin & Michel, 2009). Dam constructions led to significant change in hydrological conditions, affecting the zooplankton community (Liu et al., 2017; Liu, Liu, et al., 2019; Liu, Qin, et al., 2019). This study using DNA metabarcoding proved the seasonal and spatial differences in the community structure of zooplankton response to changes in environmental factors in the Poyang Lake Basin. Habitat variations affected by human activities and seasonal change could be the main driving factors for the variations of zooplankton community. Therefore, anthropogenic pressures need more attention in the Poyang Lake Basin.

## AUTHOR CONTRIBUTIONS

**Xuemei Xue Qiu:** Data curation (equal); formal analysis (equal); investigation (equal); methodology (equal); resources (equal); software (equal); writing – original draft (equal); writing – review and editing (equal). **Xiongjun Liu:** Data curation (equal); formal analysis (equal); funding acquisition (equal); investigation (equal); methodology (equal); resources (equal); software (equal); writing – original draft (equal); writing – review and editing (equal). **Quanfeng Lu:** Investigation (equal); resources (equal). **Jinping Chen:** Investigation (equal); resources (equal). **Tao Liang:** Investigation (equal); resources (equal). **Weikai Wang:** Investigation (equal); resources (equal). **Shan Ouyang:** Writing – original draft (equal); writing – review and editing (equal). **Chunhua Zhou:** Writing – original draft (equal); writing – review and editing (equal). **Xiaoping Wu:** Conceptualization (equal); data curation (equal); formal analysis (equal); funding acquisition (equal); project administration (equal); validation (equal); visualization (equal); writing – original draft (equal); writing – review and editing (equal).

## CONFLICT OF INTEREST

None declared.

## Supporting information

Fig S1

Fig S2

Fig S3

Table S1

Table S2

Table S3

## Data Availability

All raw sequences were deposited in the NCBI Sequence Read Archive under accession number SRA Accession no. PRJNA661399.
