# Supplementary figures and images for: Seasonal and spatial variability of zooplankton diversity in the Poyang Lake Basin using DNA metabarcoding
Source: Ecol Evol. 2022 Jun 5;12(6):e8972. doi: 10.1002/ece3.8972 (PMC9168339; doi:10.1002/ece3.8972)

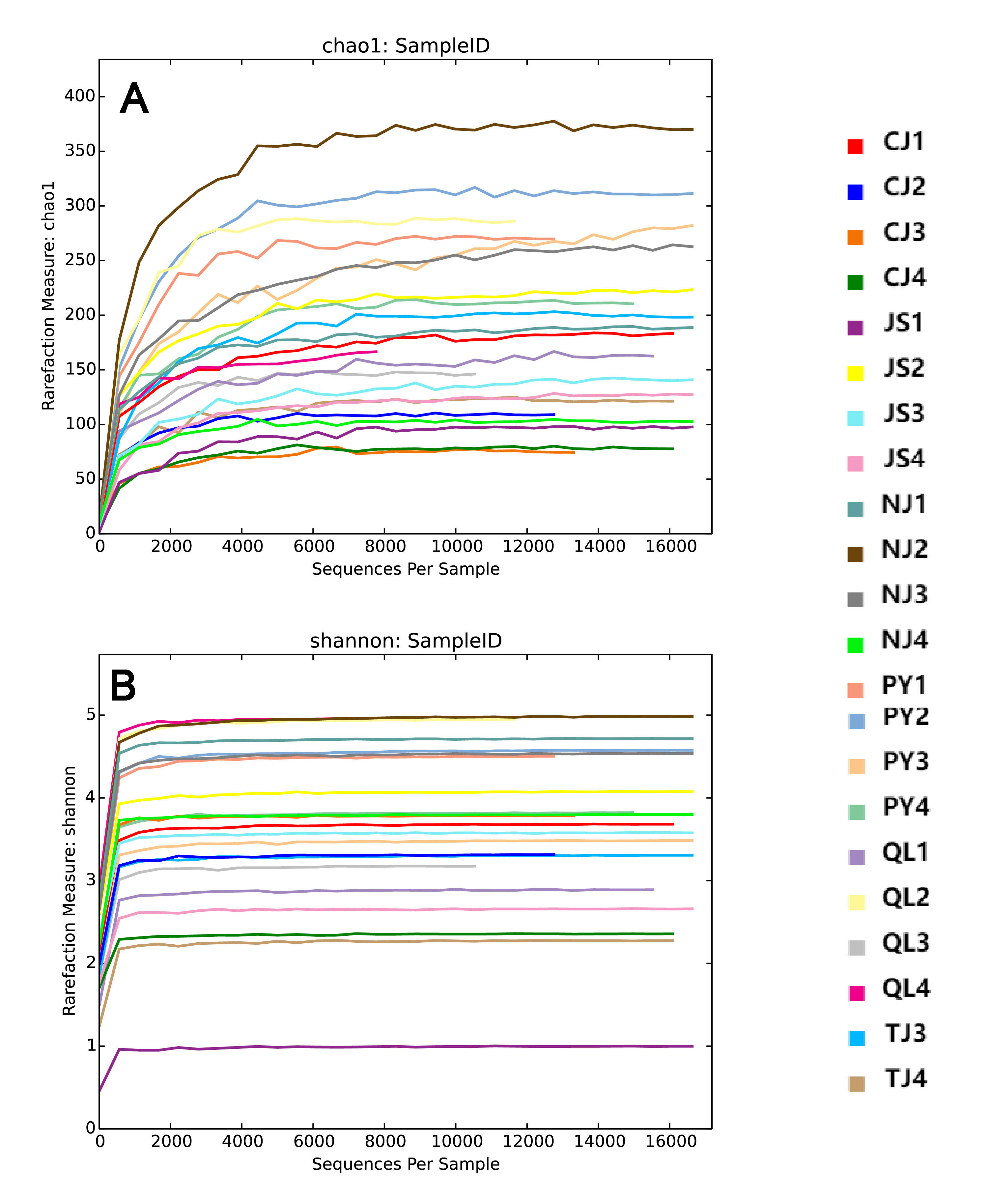

Supplement: Supplementary file 1 — Fig S1 [file ECE3-12-e8972-s005.jpg]

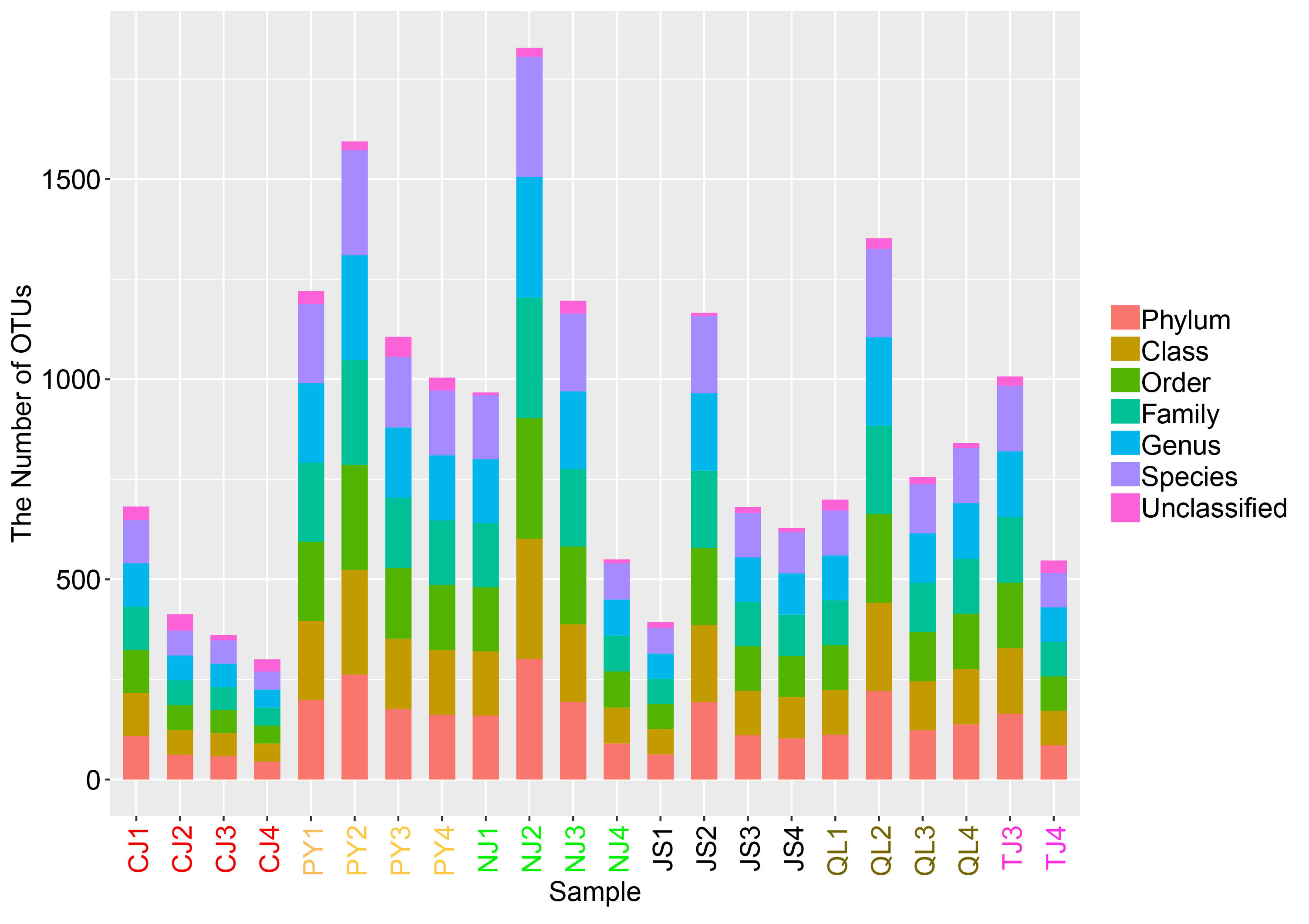

Supplement: Supplementary file 2 — Fig S2 [file ECE3-12-e8972-s004.jpg]

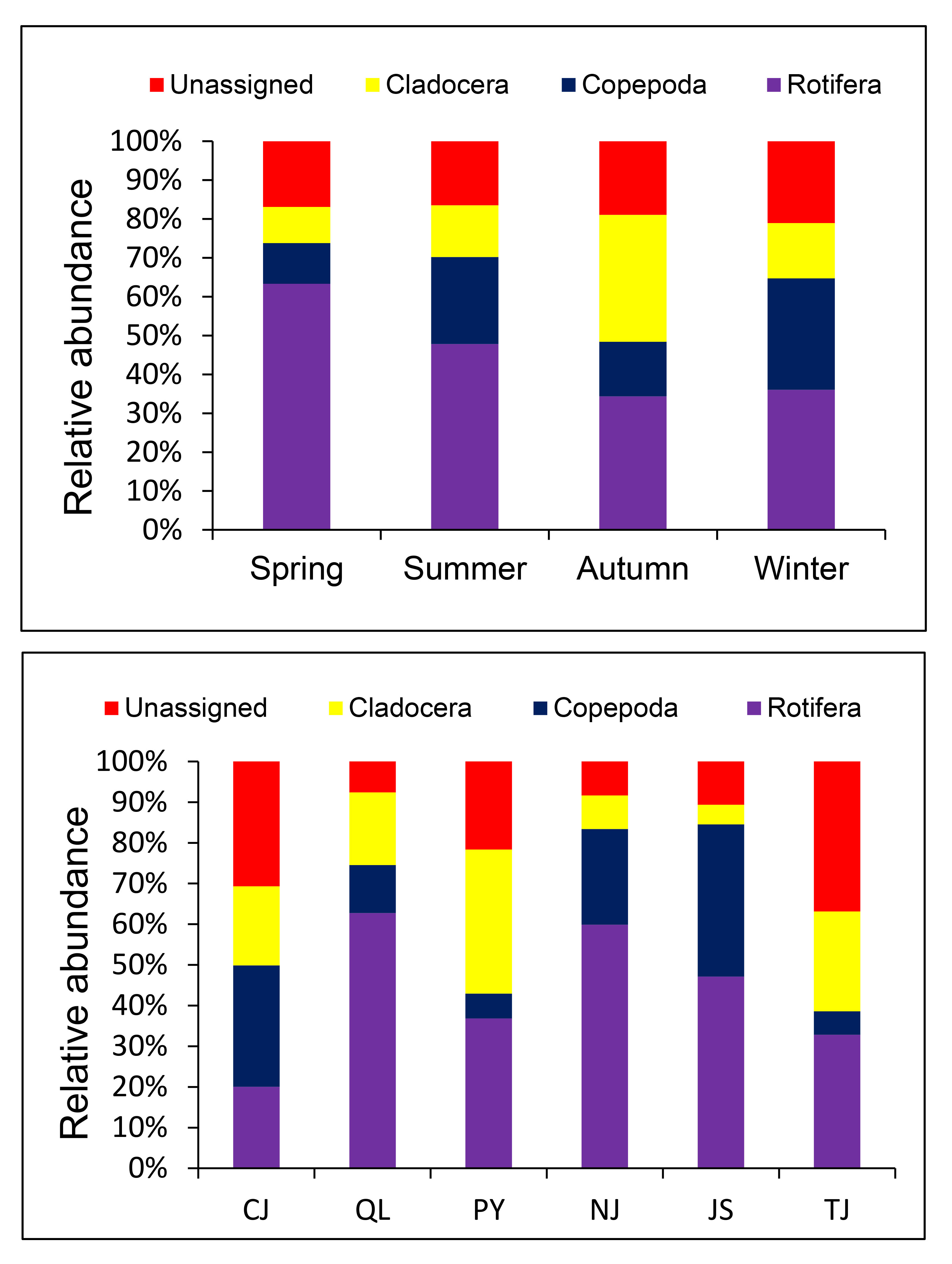

Supplement: Supplementary file 3 — Fig S3 [file ECE3-12-e8972-s001.jpg]
