# Supplementary material for: Seasonal and spatial variability of zooplankton diversity in the Poyang Lake Basin using DNA metabarcoding
Source: Ecol Evol. 2022 Jun 5;12(6):e8972. doi: 10.1002/ece3.8972 (PMC9168339; doi:10.1002/ece3.8972)
Supplement: Supplementary file 4 — Table S1 [file ECE3-12-e8972-s002.docx]

**TABLE S1.** Information of water sample collection in the Poyang Lake Basin

| Sampling areas | Time | Code | Collected water samples in the field | Samples used for DNA metabarcoding analysis |
| --- | --- | --- | --- | --- |
| Yangtze River | Spring | CJ1 | 3 (one quantitative samples of zooplankton × three samplings sites) | 1 |
|  | Summer | CJ2 | 3 (one quantitative samples of zooplankton × three samplings sites) | 1 |
|  | Autumn | CJ3 | 3 (one quantitative samples of zooplankton × three samplings sites) | 1 |
|  | Winter | CJ4 | 3 (one quantitative samples of zooplankton × three samplings sites) | 1 |
|  | Total |  |  | 4 |
| Main lake area of Poyang Lake | Spring | PY1 | 11 (one quantitative samples of zooplankton × 11 samplings sites) | 1 |
|  | Summer | PY2 | 11 (one quantitative samples of zooplankton × 11 samplings sites) | 1 |
|  | Autumn | PY3 | 11 (one quantitative samples of zooplankton × 11 samplings sites) | 1 |
|  | Winter | PY4 | 11 (one quantitative samples of zooplankton × 11 samplings sites) | 1 |
|  | Total |  |  | 4 |
| Nanjishan area of Poyang Lake | Spring | NJ1 | 5 (one quantitative samples of zooplankton × five samplings sites) | 1 |
|  | Summer | NJ2 | 5 (one quantitative samples of zooplankton × five samplings sites) | 1 |
|  | Autumn | NJ3 | 5 (one quantitative samples of zooplankton × five samplings sites) | 1 |
|  | Winter | NJ4 | 5 (one quantitative samples of zooplankton × five samplings sites) | 1 |
|  | Total |  |  | 4 |
| Junshan Lake | Spring | JS1 | 5 (one quantitative samples of zooplankton × five samplings sites) | 1 |
|  | Summer | JS2 | 5 (one quantitative samples of zooplankton × five samplings sites) | 1 |
|  | Autumn | JS3 | 5 (one quantitative samples of zooplankton × five samplings sites) | 1 |
|  | Winter | JS4 | 5 (one quantitative samples of zooplankton × five samplings sites) | 1 |
|  | Total |  |  | 4 |
| Qinglan Lake | Spring | QL1 | 5 (one quantitative samples of zooplankton × five samplings sites) | 1 |
|  | Summer | QL2 | 5 (one quantitative samples of zooplankton × five samplings sites) | 1 |
|  | Autumn | QL3 | 5 (one quantitative samples of zooplankton × five samplings sites) | 1 |
|  | Winter | QL4 | 5 (one quantitative samples of zooplankton × five samplings sites) | 1 |
|  | Total |  |  | 4 |
| Connected river channel of Poyang Lake | Autumn | TJ3 | 6 (one quantitative samples of zooplankton × six samplings sites) | 1 |
|  | Winter | TJ4 | 6 (one quantitative samples of zooplankton × six samplings sites) | 1 |
|  | Total |  |  | 2 |
