# Supplementary material for: Seasonal and spatial variability of zooplankton diversity in the Poyang Lake Basin using DNA metabarcoding
Source: Ecol Evol. 2022 Jun 5;12(6):e8972. doi: 10.1002/ece3.8972 (PMC9168339; doi:10.1002/ece3.8972)
Supplement: Supplementary file 5 — Table S2 [file ECE3-12-e8972-s006.docx]

**TABLE S2.** Seasonal and spatial changes of the total and zooplankton OTUs in Phylum, Class, Order, Family, Genus and Species from the Poyang Lake Basin. Sampling section codes are as in Table 2.

| Sample | Phylum | | Class | | Order | | Family | | Genus | | Species | | Unassigned |
| --- | --- | --- | --- | --- | --- | --- | --- | --- | --- | --- | --- | --- | --- |
|  | Total | Zooplankton | Total | Zooplankton | Total | Zooplankton | Total | Zooplankton | Total | Zooplankton | Total | Zooplankton |  |
| CJ1 | 129 | 81 | 131 | 81 | 131 | 81 | 126 | 81 | 126 | 81 | 162 | 81 | 53 |
| PY1 | 220 | 164 | 220 | 164 | 219 | 164 | 217 | 164 | 213 | 164 | 245 | 164 | 50 |
| NJ1 | 171 | 132 | 171 | 132 | 171 | 132 | 170 | 132 | 166 | 132 | 185 | 132 | 15 |
| JS1 | 68 | 37 | 68 | 37 | 68 | 37 | 58 | 37 | 58 | 37 | 79 | 37 | 20 |
| QL1 | 142 | 85 | 147 | 85 | 147 | 85 | 146 | 85 | 146 | 85 | 157 | 85 | 31 |
| CJ2 | 93 | 56 | 91 | 56 | 88 | 56 | 90 | 56 | 91 | 56 | 102 | 56 | 44 |
| PY2 | 268 | 185 | 264 | 185 | 265 | 185 | 261 | 185 | 264 | 185 | 285 | 185 | 28 |
| JS2 | 201 | 147 | 198 | 147 | 201 | 147 | 176 | 147 | 181 | 147 | 217 | 147 | 13 |
| NJ2 | 318 | 219 | 312 | 219 | 310 | 219 | 304 | 219 | 309 | 219 | 350 | 219 | 34 |
| QL2 | 235 | 186 | 230 | 186 | 231 | 186 | 213 | 186 | 220 | 186 | 264 | 186 | 44 |
| CJ3 | 72 | 48 | 75 | 48 | 79 | 48 | 79 | 48 | 78 | 48 | 85 | 48 | 14 |
| TJ3 | 183 | 109 | 181 | 109 | 182 | 109 | 181 | 109 | 179 | 109 | 186 | 109 | 12 |
| PY3 | 160 | 91 | 160 | 91 | 166 | 91 | 163 | 91 | 163 | 91 | 171 | 91 | 27 |
| JS3 | 116 | 79 | 116 | 79 | 116 | 79 | 104 | 79 | 104 | 79 | 121 | 79 | 14 |
| QL3 | 142 | 111 | 142 | 111 | 142 | 111 | 141 | 111 | 139 | 111 | 148 | 111 | 17 |
| NJ3 | 213 | 138 | 212 | 138 | 212 | 138 | 204 | 138 | 203 | 138 | 226 | 138 | 31 |
| CJ4 | 80 | 34 | 84 | 34 | 88 | 34 | 80 | 34 | 82 | 34 | 92 | 34 | 27 |
| TJ4 | 109 | 64 | 109 | 64 | 114 | 64 | 111 | 64 | 109 | 64 | 116 | 64 | 25 |
| PY4 | 156 | 124 | 157 | 124 | 161 | 124 | 156 | 124 | 154 | 124 | 168 | 124 | 24 |
| JS4 | 117 | 73 | 117 | 73 | 119 | 73 | 111 | 73 | 111 | 73 | 122 | 73 | 11 |
| QL4 | 160 | 122 | 160 | 122 | 164 | 122 | 163 | 122 | 162 | 122 | 165 | 122 | 12 |
| NJ4 | 106 | 78 | 106 | 78 | 111 | 78 | 110 | 78 | 110 | 78 | 114 | 78 | 10 |
