# Supplementary material for: Seasonal and spatial variability of zooplankton diversity in the Poyang Lake Basin using DNA metabarcoding
Source: Ecol Evol. 2022 Jun 5;12(6):e8972. doi: 10.1002/ece3.8972 (PMC9168339; doi:10.1002/ece3.8972)
Supplement: Supplementary file 6 — Table S3 [file ECE3-12-e8972-s003.docx]

**TABLE S3.** Species OTUs of zooplankton in the Poyang Lake Basin. CJ: Yangtze River; PY: Main lake area of Poyang Lake; NJ: Nanjishan area of Poyang Lake; JS: Junshan Lake; QL: Qinglan Lake; TJ: Connected river channel of Poyang Lake.

| Species | OTU ID | CJ | PY | NJ | JS | QL | TJ |
| --- | --- | --- | --- | --- | --- | --- | --- |
| *Adineta vaga* | OTU1206 | 230 | 0 | 0 | 0 | 0 | 0 |
| *Adineta vaga* | OTU2458 | 2 | 10 | 0 | 0 | 0 | 1 |
| *Adineta vaga* | OTU3450 | 12 | 0 | 0 | 0 | 0 | 0 |
| *Adineta vaga* | OTU3527 | 0 | 0 | 7 | 0 | 0 | 0 |
| *Adineta* sp. | OTU568 | 133 | 0 | 0 | 0 | 0 | 0 |
| *Asplanchna sieboldi* | OTU7554 | 0 | 10 | 0 | 0 | 31 | 27 |
| *Asplanchna silvestrii* | OTU7889 | 0 | 3 | 0 | 71 | 0 | 0 |
| *Asplanchna silvestrii* | OTU6379 | 0 | 0 | 0 | 7 | 0 | 0 |
| *Asplanchna* sp. | OTU7031 | 307 | 75 | 15 | 2 | 191 | 14 |
| *Asplanchna* sp. | OTU2802 | 0 | 4 | 63 | 0 | 500 | 0 |
| *Asplanchna* sp. | OTU431 | 0 | 0 | 101 | 0 | 0 | 0 |
| *Asplanchna* sp. | OTU4445 | 10 | 19 | 20 | 0 | 39 | 0 |
| *Asplanchna* sp. | OTU1628 | 0 | 0 | 0 | 0 | 0 | 10 |
| *Asplanchna* sp. | OTU4933 | 0 | 0 | 0 | 0 | 11 | 0 |
| *Asplanchna* sp. | OTU5616 | 0 | 5 | 0 | 0 | 0 | 0 |
| *Asplanchna* sp. | OTU6048 | 0 | 0 | 0 | 5 | 0 | 0 |
| *Asplanchna* sp. | OTU5748 | 0 | 2 | 2 | 0 | 0 | 0 |
| *Anuraeopsis* sp. | OTU6542 | 4 | 1 | 18 | 2 | 124 | 2 |
| *Brachionus Almenara* | OTU5200 | 0 | 430 | 0 | 0 | 0 | 0 |
| *Brachionus rubens* | OTU6885 | 0 | 0 | 5 | 0 | 30 | 0 |
| *Brachionus havanaensis* | OTU6195 | 9 | 0 | 0 | 0 | 0 | 0 |
| *Brachionus caudatus* | OTU94 | 39 | 0 | 0 | 0 | 0 | 3 |
| *Brachionus calyciflorus* | OTU3747 | 0 | 0 | 111 | 0 | 8 | 0 |
| *Brachionus calyciflorus* | OTU7865 | 14 | 0 | 15 | 20 | 60 | 56 |
| *Brachionus calyciflorus* | OTU2443 | 32 | 6 | 0 | 2 | 5 | 0 |
| *Brachionus calyciflorus* | OTU6577 | 0 | 3 | 0 | 3 | 23 | 0 |
| *Brachionus calyciflorus* | OTU7313 | 0 | 0 | 0 | 24 | 2 | 0 |
| *Brachionus calyciflorus* | OTU5991 | 0 | 10 | 0 | 7 | 4 | 0 |
| *Brachionus calyciflorus* | OTU1402 | 0 | 3 | 1 | 0 | 2 | 6 |
| *Brachionus calyciflorus* | OTU2169 | 0 | 0 | 0 | 0 | 39 | 0 |
| *Brachionus calyciflorus* | OTU3949 | 0 | 0 | 0 | 0 | 32 | 0 |
| *Brachionus calyciflorus* | OTU2189 | 0 | 6 | 0 | 0 | 0 | 9 |
| *Brachionus calyciflorus* | OTU8230 | 0 | 1 | 0 | 6 | 4 | 0 |
| *Brachionus calyciflorus* | OTU6049 | 0 | 0 | 0 | 0 | 13 | 0 |
| *Brachionus calyciflorus* | OTU3352 | 0 | 0 | 0 | 0 | 9 | 0 |
| *Brachionus calyciflorus* | OTU975 | 0 | 0 | 0 | 4 | 0 | 0 |
| *Brachionus quadridentatus* | OTU2400 | 0 | 2 | 1033 | 10 | 0 | 0 |
| *Brachionus quadridentatus* | OTU751 | 176 | 25 | 52 | 16 | 64 | 9 |
| *Brachionus quadridentatus* | OTU5990 | 0 | 2 | 342 | 0 | 0 | 0 |
| *Brachionus quadridentatus* | OTU6730 | 0 | 0 | 164 | 0 | 0 | 0 |
| *Brachionus quadridentatus* | OTU5480 | 0 | 0 | 86 | 0 | 0 | 0 |
| *Brachionus quadridentatus* | OTU4319 | 0 | 13 | 6 | 10 | 0 | 23 |
| *Brachionus quadridentatus* | OTU1595 | 0 | 0 | 34 | 0 | 0 | 0 |
| *Brachionus quadridentatus* | OTU2212 | 0 | 0 | 29 | 0 | 0 | 0 |
| *Brachionus quadridentatus* | OTU2011 | 0 | 10 | 0 | 13 | 1 | 0 |
| *Brachionus quadridentatus* | OTU5288 | 0 | 2 | 2 | 4 | 13 | 0 |
| *Brachionus quadridentatus* | OTU2188 | 0 | 0 | 14 | 0 | 0 | 0 |
| *Brachionus quadridentatus* | OTU685 | 0 | 0 | 0 | 9 | 0 | 0 |
| *Brachionus quadridentatus* | OTU3405 | 0 | 0 | 8 | 0 | 0 | 0 |
| *Brachionus quadridentatus* | OTU395 | 0 | 0 | 5 | 0 | 0 | 0 |
| *Brachionus quadridentatus* | OTU2889 | 0 | 0 | 5 | 0 | 0 | 0 |
| *Brachionus quadridentatus* | OTU41 | 0 | 0 | 0 | 0 | 5 | 0 |
| *Brachionus quadridentatus* | OTU2083 | 0 | 0 | 0 | 0 | 2 | 0 |
| *Brachionus quadridentatus* | OTU6466 | 0 | 0 | 0 | 0 | 3 | 0 |
| *Brachionus quadridentatus* | OTU797 | 0 | 0 | 4 | 0 | 0 | 0 |
| *Brachionus urceolaris* | OTU8313 | 63 | 40 | 112 | 59 | 72 | 13 |
| *Brachionus urceolaris* | OTU4372 | 0 | 0 | 75 | 0 | 0 | 0 |
| *Brachionus urceolaris* | OTU900 | 0 | 0 | 22 | 0 | 0 | 0 |
| *Brachionus urceolaris* | OTU1300 | 0 | 0 | 2 | 9 | 0 | 0 |
| *Brachionus urceolaris* | OTU2855 | 0 | 5 | 0 | 2 | 0 | 0 |
| *Brachionus urceolaris* | OTU113 | 0 | 0 | 4 | 0 | 1 | 0 |
| *Brachionus urceolaris* | OTU5848 | 0 | 0 | 0 | 4 | 0 | 0 |
| *Brachionus urceolaris* | OTU1963 | 0 | 0 | 2 | 0 | 0 | 0 |
| *Brachionus* sp. | OTU4070 | 533 | 1362 | 1420 | 1207 | 10569 | 147 |
| *Brachionus* sp. | OTU5344 | 2091 | 66 | 99 | 15 | 39 | 32 |
| *Brachionus* sp. | OTU2699 | 16 | 6 | 470 | 0 | 0 | 13 |
| *Brachionus* sp. | OTU6589 | 0 | 0 | 538 | 0 | 23 | 0 |
| *Brachionus* sp. | OTU6428 | 355 | 28 | 4 | 6 | 87 | 3 |
| *Brachionus* sp. | OTU2616 | 0 | 0 | 319 | 0 | 0 | 0 |
| *Brachionus* sp. | OTU1850 | 206 | 25 | 5 | 28 | 21 | 0 |
| *Brachionus* sp. | OTU3082 | 1 | 2 | 168 | 8 | 3 | 0 |
| *Brachionus* sp. | OTU4766 | 0 | 0 | 150 | 0 | 0 | 0 |
| *Brachionus* sp. | OTU6020 | 19 | 1 | 52 | 0 | 0 | 25 |
| *Brachionus* sp. | OTU398 | 179 | 0 | 0 | 0 | 0 | 0 |
| *Brachionus* sp. | OTU6683 | 76 | 13 | 4 | 23 | 8 | 0 |
| *Brachionus* sp. | OTU312 | 0 | 28 | 0 | 7 | 0 | 0 |
| *Brachionus* sp. | OTU6259 | 0 | 1 | 0 | 20 | 2 | 0 |
| *Brachionus* sp. | OTU5484 | 0 | 0 | 42 | 0 | 0 | 0 |
| *Brachionus* sp. | OTU563 | 0 | 7 | 0 | 4 | 8 | 0 |
| *Brachionus* sp. | OTU7662 | 0 | 0 | 3 | 0 | 21 | 0 |
| *Brachionus* sp. | OTU8307 | 0 | 0 | 22 | 0 | 0 | 0 |
| *Brachionus* sp. | OTU5956 | 0 | 4 | 17 | 0 | 0 | 0 |
| *Brachionus* sp. | OTU4782 | 0 | 0 | 0 | 2 | 19 | 0 |
| *Brachionus* sp. | OTU6399 | 0 | 0 | 19 | 0 | 0 | 0 |
| *Brachionus* sp. | OTU1494 | 0 | 0 | 19 | 0 | 0 | 0 |
| *Brachionus* sp. | OTU7078 | 0 | 0 | 0 | 0 | 20 | 0 |
| *Brachionus* sp. | OTU7506 | 0 | 3 | 4 | 4 | 6 | 0 |
| *Brachionus* sp. | OTU7219 | 0 | 0 | 11 | 0 | 0 | 0 |
| *Brachionus* sp. | OTU5688 | 0 | 4 | 1 | 4 | 4 | 0 |
| *Brachionus* sp. | OTU4698 | 6 | 2 | 0 | 5 | 2 | 0 |
| *Brachionus* sp. | OTU4090 | 0 | 0 | 10 | 1 | 0 | 0 |
| *Brachionus* sp. | OTU4927 | 0 | 0 | 11 | 0 | 0 | 0 |
| *Brachionus* sp. | OTU7770 | 0 | 0 | 9 | 0 | 0 | 0 |
| *Brachionus* sp. | OTU2857 | 0 | 9 | 3 | 0 | 0 | 0 |
| *Brachionus* sp. | OTU7316 | 0 | 2 | 0 | 2 | 2 | 0 |
| *Brachionus* sp. | OTU6189 | 0 | 2 | 0 | 0 | 0 | 0 |
| *Brachionus* sp. | OTU965 | 0 | 0 | 7 | 0 | 0 | 0 |
| *Brachionus* sp. | OTU1313 | 0 | 0 | 7 | 0 | 0 | 0 |
| *Brachionus* sp. | OTU3891 | 0 | 0 | 6 | 0 | 0 | 0 |
| *Brachionus* sp. | OTU1528 | 0 | 0 | 6 | 0 | 0 | 0 |
| *Brachionus* sp. | OTU8181 | 0 | 0 | 5 | 0 | 0 | 0 |
| *Brachionus* sp. | OTU905 | 0 | 1 | 0 | 5 | 0 | 0 |
| *Brachionus* sp. | OTU1746 | 0 | 0 | 0 | 5 | 0 | 0 |
| *Brachionus* sp. | OTU7641 | 0 | 4 | 0 | 1 | 0 | 0 |
| *Brachionus* sp. | OTU2264 | 0 | 3 | 0 | 1 | 1 | 0 |
| *Brachionus* sp. | OTU4806 | 0 | 4 | 0 | 0 | 0 | 0 |
| *Brachionus* sp. | OTU7026 | 0 | 2 | 1 | 0 | 1 | 0 |
| *Brachionus* sp. | OTU2357 | 0 | 4 | 0 | 0 | 0 | 0 |
| *Brachionus* sp. | OTU4124 | 0 | 2 | 0 | 2 | 0 | 0 |
| *Brachionus* sp. | OTU7841 | 0 | 4 | 0 | 0 | 0 | 0 |
| *Brachionus* sp. | OTU6810 | 0 | 0 | 4 | 0 | 0 | 0 |
| *Brachionus* sp. | OTU2134 | 0 | 0 | 4 | 0 | 0 | 0 |
| *Brachionus* sp. | OTU4233 | 0 | 0 | 5 | 0 | 0 | 0 |
| *Brachionus* sp. | OTU7791 | 0 | 0 | 0 | 2 | 0 | 0 |
| *Brachionus* sp. | OTU2490 | 0 | 1 | 0 | 1 | 1 | 0 |
| *Brachionus* sp. | OTU2894 | 0 | 2 | 0 | 0 | 0 | 0 |
| *Brachionus* sp. | OTU3053 | 0 | 2 | 0 | 2 | 0 | 0 |
| *Brachionus* sp. | OTU4347 | 0 | 0 | 2 | 0 | 0 | 0 |
| *Brachionus* sp. | OTU5556 | 0 | 0 | 4 | 0 | 0 | 0 |
| *Brachionus plicatilis* | OTU1579 | 45 | 10 | 3433 | 15 | 38 | 0 |
| *Brachionus plicatilis* | OTU2663 | 0 | 6 | 2185 | 19 | 40 | 0 |
| *Brachionus plicatilis* | OTU8477 | 28 | 4 | 1231 | 0 | 0 | 0 |
| *Brachionus plicatilis* | OTU2772 | 0 | 6 | 282 | 4 | 0 | 0 |
| *Brachionus plicatilis* | OTU1960 | 0 | 3 | 250 | 0 | 1 | 0 |
| *Brachionus plicatilis* | OTU4940 | 0 | 0 | 200 | 0 | 0 | 0 |
| *Brachionus plicatilis* | OTU7752 | 0 | 0 | 147 | 0 | 0 | 0 |
| *Brachionus plicatilis* | OTU4563 | 0 | 0 | 98 | 3 | 0 | 0 |
| *Brachionus plicatilis* | OTU5889 | 0 | 0 | 51 | 0 | 0 | 0 |
| *Brachionus plicatilis* | OTU140 | 0 | 0 | 46 | 0 | 0 | 0 |
| *Brachionus plicatilis* | OTU7407 | 0 | 0 | 44 | 0 | 0 | 0 |
| *Brachionus plicatilis* | OTU5180 | 0 | 0 | 39 | 0 | 1 | 0 |
| *Brachionus plicatilis* | OTU1740 | 0 | 0 | 30 | 0 | 0 | 0 |
| *Brachionus plicatilis* | OTU8393 | 0 | 0 | 20 | 0 | 0 | 0 |
| *Brachionus plicatilis* | OTU3310 | 0 | 0 | 19 | 0 | 0 | 0 |
| *Brachionus plicatilis* | OTU8097 | 0 | 0 | 20 | 0 | 0 | 0 |
| *Brachionus plicatilis* | OTU5426 | 0 | 0 | 15 | 0 | 0 | 0 |
| *Brachionus plicatilis* | OTU4704 | 0 | 0 | 13 | 0 | 0 | 0 |
| *Brachionus plicatilis* | OTU353 | 0 | 0 | 0 | 10 | 0 | 0 |
| *Brachionus plicatilis* | OTU8470 | 0 | 0 | 9 | 0 | 0 | 0 |
| *Brachionus plicatilis* | OTU1483 | 0 | 0 | 0 | 5 | 2 | 0 |
| *Brachionus plicatilis* | OTU2278 | 0 | 0 | 8 | 0 | 0 | 0 |
| *Brachionus plicatilis* | OTU2691 | 0 | 0 | 5 | 0 | 0 | 0 |
| *Brachionus plicatilis* | OTU4778 | 0 | 0 | 5 | 0 | 0 | 0 |
| *Brachionus plicatilis* | OTU7053 | 0 | 0 | 4 | 0 | 0 | 0 |
| *Brachionus plicatilis* | OTU2629 | 0 | 3 | 0 | 0 | 0 | 0 |
| *Brachionus plicatilis* | OTU5970 | 0 | 0 | 4 | 0 | 0 | 0 |
| *Brachionus plicatilis* | OTU7798 | 0 | 0 | 4 | 0 | 0 | 0 |
| Brachionidae sp. | OTU4503 | 0 | 0 | 152 | 0 | 1 | 8 |
| Brachionidae sp. | OTU8040 | 0 | 0 | 0 | 0 | 111 | 0 |
| Brachionidae sp. | OTU3863 | 11 | 0 | 78 | 0 | 1 | 8 |
| Brachionidae sp. | OTU4497 | 0 | 8 | 18 | 11 | 24 | 0 |
| Brachionidae sp. | OTU7672 | 0 | 0 | 0 | 15 | 0 | 0 |
| Brachionidae sp. | OTU2524 | 0 | 0 | 0 | 0 | 27 | 0 |
| Brachionidae sp. | OTU4970 | 0 | 0 | 7 | 0 | 0 | 0 |
| *Euchlanis dilatata* | OTU7991 | 0 | 492 | 16 | 95 | 90 | 2 |
| *Euchlanis dilatata* | OTU3835 | 0 | 3 | 0 | 0 | 447 | 0 |
| *Euchlanis dilatata* | OTU5992 | 0 | 74 | 20 | 41 | 0 | 194 |
| *Euchlanis dilatata* | OTU1865 | 0 | 9 | 2 | 0 | 321 | 4 |
| *Euchlanis dilatata* | OTU5406 | 0 | 0 | 0 | 0 | 148 | 3 |
| *Euchlanis dilatata* | OTU2459 | 0 | 70 | 8 | 22 | 5 | 9 |
| *Euchlanis dilatata* | OTU7155 | 0 | 2 | 0 | 0 | 2 | 102 |
| *Euchlanis dilatata* | OTU8127 | 0 | 19 | 0 | 14 | 0 | 0 |
| *Euchlanis dilatata* | OTU3723 | 5 | 7 | 1 | 3 | 0 | 5 |
| *Euchlanis dilatata* | OTU6545 | 0 | 4 | 0 | 0 | 22 | 0 |
| *Euchlanis dilatata* | OTU886 | 0 | 1 | 1 | 12 | 0 | 0 |
| *Euchlanis dilatata* | OTU3207 | 0 | 4 | 14 | 0 | 0 | 0 |
| *Euchlanis dilatata* | OTU3848 | 0 | 0 | 0 | 0 | 14 | 0 |
| *Euchlanis dilatata* | OTU2030 | 0 | 0 | 0 | 0 | 18 | 0 |
| *Euchlanis dilatata* | OTU1874 | 0 | 11 | 0 | 0 | 6 | 0 |
| *Euchlanis dilatata* | OTU6917 | 0 | 0 | 10 | 0 | 16 | 0 |
| *Euchlanis dilatata* | OTU4495 | 0 | 1 | 3 | 8 | 0 | 0 |
| *Euchlanis dilatata* | OTU6155 | 0 | 5 | 0 | 5 | 0 | 0 |
| *Euchlanis dilatata* | OTU3565 | 0 | 2 | 0 | 9 | 0 | 0 |
| *Euchlanis dilatata* | OTU1950 | 0 | 7 | 0 | 2 | 0 | 0 |
| *Euchlanis dilatata* | OTU1832 | 0 | 0 | 0 | 0 | 8 | 0 |
| *Euchlanis dilatata* | OTU2946 | 0 | 5 | 0 | 1 | 2 | 0 |
| *Euchlanis dilatata* | OTU982 | 0 | 3 | 0 | 3 | 0 | 0 |
| *Euchlanis dilatata* | OTU2826 | 1 | 0 | 5 | 0 | 0 | 0 |
| *Euchlanis dilatata* | OTU8123 | 0 | 2 | 0 | 3 | 0 | 0 |
| *Euchlanis dilatata* | OTU246 | 0 | 0 | 0 | 0 | 5 | 0 |
| *Euchlanis dilatata* | OTU7718 | 0 | 0 | 0 | 0 | 4 | 0 |
| *Euchlanis dilatata* | OTU6606 | 0 | 0 | 0 | 0 | 4 | 0 |
| *Keratella cochlearis* | OTU5262 | 0 | 20 | 0 | 8 | 4 | 0 |
| *Keratella cochlearis* | OTU1371 | 0 | 14 | 0 | 4 | 5 | 0 |
| *Keratella cochlearis* | OTU7856 | 0 | 12 | 0 | 3 | 2 | 0 |
| *Keratella cochlearis* | OTU5906 | 0 | 5 | 0 | 2 | 4 | 0 |
| *Keratella cochlearis* | OTU2044 | 0 | 0 | 0 | 5 | 0 | 1 |
| *Keratella cochlearis* | OTU5467 | 0 | 5 | 0 | 2 | 0 | 0 |
| *Keratella cochlearis* | OTU2694 | 1 | 3 | 0 | 2 | 0 | 0 |
| *Keratella cochlearis* | OTU6004 | 0 | 3 | 0 | 2 | 0 | 0 |
| *Keratella cochlearis* | OTU2327 | 0 | 1 | 0 | 2 | 0 | 0 |
| *Keratella* sp. | OTU1211 | 226 | 253 | 6445 | 1072 | 451 | 249 |
| *Keratella* sp. | OTU1123 | 345 | 788 | 4049 | 622 | 1526 | 1270 |
| *Keratella* sp. | OTU1802 | 244 | 781 | 1078 | 690 | 651 | 157 |
| *Keratella* sp. | OTU7343 | 326 | 204 | 796 | 80 | 402 | 166 |
| *Keratella* sp. | OTU7924 | 433 | 68 | 330 | 17 | 87 | 142 |
| *Keratella* sp. | OTU4945 | 63 | 65 | 261 | 80 | 75 | 102 |
| *Keratella* sp. | OTU6481 | 180 | 36 | 232 | 21 | 191 | 33 |
| *Keratella* sp. | OTU5377 | 25 | 100 | 170 | 96 | 64 | 45 |
| *Keratella* sp. | OTU3615 | 0 | 8 | 193 | 120 | 116 | 28 |
| *Keratella* sp. | OTU2896 | 0 | 31 | 37 | 16 | 157 | 12 |
| *Keratella* sp. | OTU480 | 1 | 24 | 118 | 15 | 13 | 25 |
| *Keratella* sp. | OTU155 | 90 | 7 | 12 | 2 | 22 | 0 |
| *Keratella* sp. | OTU7530 | 33 | 4 | 11 | 0 | 46 | 8 |
| *Keratella* sp. | OTU6475 | 4 | 11 | 42 | 6 | 15 | 9 |
| *Keratella* sp. | OTU1233 | 0 | 2 | 10 | 2 | 94 | 2 |
| *Keratella* sp. | OTU5369 | 0 | 0 | 43 | 1 | 3 | 5 |
| *Keratella* sp. | OTU2178 | 0 | 0 | 45 | 2 | 0 | 0 |
| *Keratella* sp. | OTU6056 | 0 | 7 | 15 | 5 | 4 | 22 |
| *Keratella* sp. | OTU3144 | 2 | 0 | 31 | 2 | 4 | 2 |
| *Keratella* sp. | OTU7185 | 0 | 3 | 31 | 4 | 1 | 3 |
| *Keratella* sp. | OTU5253 | 0 | 0 | 38 | 0 | 0 | 0 |
| *Keratella* sp. | OTU6303 | 1 | 8 | 14 | 5 | 7 | 4 |
| *Keratella* sp. | OTU2957 | 0 | 6 | 0 | 5 | 0 | 12 |
| *Keratella* sp. | OTU6849 | 0 | 0 | 41 | 0 | 0 | 3 |
| *Keratella* sp. | OTU6518 | 0 | 0 | 0 | 7 | 17 | 0 |
| *Keratella* sp. | OTU4915 | 0 | 0 | 15 | 4 | 0 | 3 |
| *Keratella* sp. | OTU5977 | 2 | 0 | 11 | 1 | 4 | 2 |
| *Keratella* sp. | OTU5323 | 0 | 0 | 0 | 0 | 0 | 15 |
| *Keratella* sp. | OTU1351 | 0 | 6 | 9 | 1 | 0 | 2 |
| *Keratella* sp. | OTU5105 | 0 | 6 | 12 | 0 | 1 | 0 |
| *Keratella* sp. | OTU6034 | 0 | 6 | 13 | 0 | 0 | 0 |
| *Keratella* sp. | OTU5639 | 0 | 6 | 0 | 4 | 0 | 6 |
| *Keratella* sp. | OTU4772 | 0 | 8 | 5 | 0 | 2 | 2 |
| *Keratella* sp. | OTU7149 | 0 | 0 | 7 | 3 | 6 | 1 |
| *Keratella* sp. | OTU3941 | 0 | 2 | 6 | 0 | 4 | 2 |
| *Keratella* sp. | OTU7461 | 0 | 0 | 17 | 0 | 0 | 0 |
| *Keratella* sp. | OTU153 | 0 | 17 | 0 | 0 | 0 | 4 |
| *Keratella* sp. | OTU3124 | 1 | 0 | 5 | 1 | 3 | 4 |
| *Keratella* sp. | OTU2383 | 0 | 0 | 11 | 0 | 0 | 0 |
| *Keratella* sp. | OTU4209 | 0 | 0 | 5 | 0 | 3 | 3 |
| *Keratella* sp. | OTU6511 | 0 | 3 | 5 | 0 | 2 | 0 |
| *Keratella* sp. | OTU7839 | 0 | 8 | 2 | 3 | 0 | 0 |
| *Keratella* sp. | OTU186 | 0 | 1 | 5 | 2 | 1 | 0 |
| *Keratella* sp. | OTU3616 | 0 | 4 | 0 | 5 | 0 | 0 |
| *Keratella* sp. | OTU5609 | 0 | 0 | 3 | 4 | 2 | 0 |
| *Keratella* sp. | OTU4759 | 0 | 4 | 5 | 1 | 0 | 0 |
| *Keratella* sp. | OTU6564 | 0 | 1 | 7 | 1 | 1 | 2 |
| *Keratella* sp. | OTU791 | 0 | 0 | 0 | 7 | 0 | 0 |
| *Keratella* sp. | OTU2256 | 0 | 2 | 5 | 0 | 2 | 0 |
| *Keratella* sp. | OTU3219 | 0 | 0 | 5 | 0 | 1 | 2 |
| *Keratella* sp. | OTU4906 | 0 | 0 | 4 | 0 | 1 | 1 |
| *Keratella* sp. | OTU1084 | 0 | 0 | 2 | 1 | 3 | 1 |
| *Keratella* sp. | OTU1088 | 0 | 0 | 5 | 0 | 0 | 0 |
| *Keratella* sp. | OTU7996 | 0 | 0 | 8 | 0 | 1 | 0 |
| *Keratella* sp. | OTU8045 | 0 | 1 | 3 | 2 | 2 | 0 |
| *Keratella* sp. | OTU1455 | 3 | 0 | 3 | 0 | 0 | 1 |
| *Keratella* sp. | OTU3620 | 0 | 0 | 3 | 0 | 0 | 0 |
| *Keratella* sp. | OTU2677 | 0 | 0 | 5 | 0 | 0 | 0 |
| *Keratella* sp. | OTU7486 | 0 | 0 | 2 | 3 | 2 | 0 |
| *Keratella* sp. | OTU1223 | 0 | 0 | 4 | 0 | 0 | 0 |
| *Keratella* sp. | OTU3563 | 0 | 1 | 0 | 1 | 3 | 0 |
| *Keratella* sp. | OTU2637 | 0 | 1 | 0 | 3 | 0 | 0 |
| *Keratella* sp. | OTU4079 | 0 | 2 | 0 | 2 | 0 | 0 |
| *Keratella* sp. | OTU5568 | 1 | 2 | 0 | 0 | 0 | 0 |
| *Keratella* sp. | OTU2455 | 0 | 0 | 4 | 0 | 0 | 0 |
| *Keratella* sp. | OTU3244 | 0 | 0 | 2 | 2 | 0 | 0 |
| *Keratella* sp. | OTU5702 | 0 | 0 | 5 | 0 | 0 | 0 |
| *Keratella* sp. | OTU7101 | 0 | 0 | 3 | 0 | 0 | 0 |
| *Keratella* sp. | OTU231 | 0 | 2 | 0 | 0 | 1 | 0 |
| *Keratella* sp. | OTU287 | 0 | 0 | 0 | 0 | 1 | 0 |
| *Keratella* sp. | OTU4893 | 0 | 0 | 2 | 0 | 0 | 0 |
| *Keratella* sp. | OTU7799 | 0 | 0 | 2 | 0 | 0 | 0 |
| *Plationus patulus* | OTU995 | 39 | 0 | 0 | 0 | 0 | 0 |
| *Plationus patulus* | OTU4040 | 0 | 0 | 25 | 0 | 8 | 0 |
| *Plationus patulus* | OTU1413 | 0 | 0 | 1 | 15 | 0 | 0 |
| *Plationus patulus* | OTU708 | 0 | 0 | 0 | 2 | 0 | 7 |
| *Plationus patulus* | OTU753 | 0 | 0 | 6 | 0 | 0 | 0 |
| *Plationus patulus* | OTU679 | 0 | 2 | 0 | 1 | 0 | 0 |
| *Plationus patulus* | OTU2584 | 0 | 0 | 0 | 3 | 0 | 0 |
| *Plationus patulus* | OTU2463 | 0 | 0 | 0 | 1 | 2 | 0 |
| *Plationus patulus* | OTU2846 | 0 | 0 | 0 | 0 | 2 | 0 |
| *Conochiloides* sp. | OTU6199 | 0 | 0 | 0 | 0 | 13 | 0 |
| *Lecane* sp. | OTU1936 | 0 | 4 | 921 | 2 | 1 | 0 |
| *Lecane* sp. | OTU1831 | 0 | 63 | 26 | 190 | 68 | 7 |
| *Lecane* sp. | OTU6164 | 33 | 24 | 100 | 56 | 4 | 117 |
| *Lecane* sp. | OTU8206 | 0 | 8 | 7 | 10 | 2 | 18 |
| *Lecane* sp. | OTU4317 | 0 | 5 | 2 | 16 | 8 | 0 |
| *Lecane* sp. | OTU6451 | 45 | 0 | 0 | 0 | 0 | 0 |
| *Lecane* sp. | OTU2822 | 0 | 2 | 0 | 5 | 0 | 0 |
| *Lecane* sp. | OTU1909 | 0 | 0 | 0 | 3 | 1 | 0 |
| *Lecane* sp. | OTU512 | 0 | 0 | 0 | 0 | 0 | 2 |
| *Lecane* sp. | OTU5794 | 0 | 0 | 4 | 1 | 0 | 0 |
| *Lecane* sp. | OTU766 | 0 | 1 | 0 | 2 | 0 | 0 |
| *Lecane ohioensis* | OTU7751 | 2900 | 4085 | 828 | 4100 | 2045 | 6406 |
| *Lecane ohioensis* | OTU7766 | 0 | 636 | 105 | 718 | 145 | 306 |
| *Lecane ohioensis* | OTU851 | 0 | 177 | 84 | 24 | 224 | 0 |
| *Lecane ohioensis* | OTU2759 | 0 | 64 | 1 | 95 | 160 | 14 |
| *Lecane ohioensis* | OTU7527 | 0 | 126 | 30 | 68 | 23 | 47 |
| *Lecane ohioensis* | OTU6256 | 0 | 54 | 17 | 35 | 10 | 69 |
| *Lecane ohioensis* | OTU7536 | 0 | 135 | 21 | 40 | 0 | 5 |
| *Lecane ohioensis* | OTU6255 | 0 | 9 | 4 | 13 | 0 | 43 |
| *Lecane ohioensis* | OTU6046 | 0 | 1 | 20 | 33 | 0 | 0 |
| *Lecane ohioensis* | OTU3333 | 0 | 18 | 0 | 17 | 0 | 9 |
| *Lecane ohioensis* | OTU5709 | 0 | 25 | 0 | 17 | 0 | 0 |
| *Lecane ohioensis* | OTU3950 | 0 | 13 | 0 | 0 | 23 | 0 |
| *Lecane ohioensis* | OTU2607 | 0 | 20 | 0 | 4 | 6 | 0 |
| *Lecane ohioensis* | OTU4694 | 0 | 14 | 2 | 6 | 0 | 4 |
| *Lecane ohioensis* | OTU631 | 0 | 7 | 2 | 4 | 0 | 2 |
| *Lecane ohioensis* | OTU6628 | 0 | 12 | 0 | 2 | 1 | 0 |
| *Lecane ohioensis* | OTU329 | 0 | 4 | 0 | 5 | 4 | 0 |
| *Lecane ohioensis* | OTU6792 | 2 | 4 | 0 | 4 | 2 | 0 |
| *Lecane ohioensis* | OTU1550 | 0 | 4 | 0 | 3 | 0 | 2 |
| *Lecane ohioensis* | OTU5300 | 0 | 5 | 0 | 3 | 0 | 2 |
| *Lecane ohioensis* | OTU2704 | 0 | 4 | 0 | 6 | 0 | 0 |
| *Lecane ohioensis* | OTU943 | 0 | 3 | 1 | 6 | 0 | 0 |
| *Lecane ohioensis* | OTU2948 | 0 | 4 | 0 | 6 | 0 | 0 |
| *Lecane ohioensis* | OTU1329 | 2 | 2 | 1 | 3 | 0 | 1 |
| *Lecane ohioensis* | OTU5475 | 0 | 7 | 1 | 1 | 0 | 1 |
| *Lecane ohioensis* | OTU874 | 0 | 6 | 0 | 0 | 0 | 0 |
| *Lecane ohioensis* | OTU4353 | 2 | 0 | 5 | 0 | 0 | 0 |
| *Lecane ohioensis* | OTU2446 | 0 | 0 | 0 | 5 | 0 | 1 |
| *Lecane ohioensis* | OTU775 | 0 | 4 | 0 | 1 | 1 | 0 |
| *Lecane ohioensis* | OTU8471 | 0 | 5 | 0 | 2 | 1 | 0 |
| *Lecane ohioensis* | OTU452 | 0 | 4 | 0 | 0 | 0 | 0 |
| *Lecane ohioensis* | OTU5893 | 0 | 0 | 6 | 0 | 0 | 0 |
| *Lecane ohioensis* | OTU6071 | 0 | 3 | 0 | 1 | 0 | 0 |
| *Lecane ohioensis* | OTU1567 | 0 | 2 | 0 | 2 | 0 | 0 |
| *Lecane ohioensis* | OTU5511 | 0 | 3 | 0 | 0 | 0 | 0 |
| *Lecane cornuta* | OTU4988 | 8 | 0 | 0 | 0 | 0 | 0 |
| *Lecane cornuta* | OTU1336 | 0 | 0 | 0 | 4 | 0 | 0 |
| *Lecane closterocerca* | OTU4156 | 0 | 905 | 4 | 126 | 903 | 0 |
| *Lecane closterocerca* | OTU3776 | 0 | 11 | 0 | 1 | 18 | 0 |
| *Lecane closterocerca* | OTU8049 | 0 | 20 | 0 | 0 | 0 | 0 |
| *Lecane closterocerca* | OTU7005 | 0 | 11 | 0 | 3 | 2 | 0 |
| *Lecane closterocerca* | OTU2925 | 0 | 3 | 0 | 0 | 7 | 0 |
| *Lecane closterocerca* | OTU1338 | 0 | 6 | 0 | 0 | 3 | 0 |
| *Lecane closterocerca* | OTU7880 | 5 | 0 | 3 | 0 | 1 | 0 |
| *Lecane closterocerca* | OTU4763 | 0 | 2 | 0 | 0 | 5 | 0 |
| *Lecane closterocerca* | OTU4210 | 0 | 4 | 0 | 0 | 3 | 0 |
| *Lecane closterocerca* | OTU6713 | 0 | 3 | 0 | 0 | 3 | 0 |
| *Lecane closterocerca* | OTU579 | 0 | 3 | 0 | 0 | 2 | 0 |
| *Lecane closterocerca* | OTU3121 | 0 | 2 | 0 | 0 | 3 | 0 |
| *Lecane closterocerca* | OTU6103 | 0 | 2 | 0 | 0 | 2 | 0 |
| *Lecane closterocerca* | OTU1988 | 0 | 2 | 0 | 0 | 2 | 0 |
| *Lecane closterocerca* | OTU3029 | 0 | 2 | 0 | 0 | 1 | 0 |
| *Lecane closterocerca* | OTU8220 | 0 | 1 | 0 | 0 | 2 | 0 |
| *Lecane closterocerca* | OTU3230 | 0 | 2 | 0 | 0 | 0 | 0 |
| *Lecane curvicornis* | OTU2625 | 25 | 0 | 0 | 0 | 0 | 0 |
| *Lecane lunaris* | OTU367 | 0 | 4 | 0 | 1 | 0 | 0 |
| *Lecane bulla* | OTU677 | 0 | 0 | 0 | 0 | 16 | 0 |
| *Lecane papuana* | OTU6478 | 0 | 0 | 0 | 0 | 32 | 0 |
| Lecanidae sp. | OTU8502 | 0 | 0 | 10 | 0 | 3 | 3 |
| Lepadellidae sp. | OTU1654 | 0 | 0 | 0 | 0 | 22 | 0 |
| Lepadellidae sp. | OTU2101 | 0 | 0 | 17 | 0 | 0 | 0 |
| Lepadellidae sp. | OTU4020 | 0 | 0 | 11 | 0 | 0 | 0 |
| Lepadellidae sp. | OTU2350 | 0 | 0 | 9 | 0 | 0 | 0 |
| Lepadellidae sp. | OTU4686 | 0 | 0 | 7 | 0 | 0 | 0 |
| *Filinia* sp. | OTU1275 | 89 | 89 | 51 | 27 | 172 | 15 |
| *Filinia* sp. | OTU3024 | 9 | 24 | 155 | 4 | 22 | 3 |
| *Filinia* sp. | OTU3064 | 0 | 1 | 2 | 8 | 205 | 0 |
| *Filinia* sp. | OTU2433 | 28 | 0 | 0 | 2 | 13 | 0 |
| *Filinia* sp. | OTU1612 | 0 | 3 | 11 | 0 | 28 | 0 |
| *Filinia* sp. | OTU5929 | 0 | 0 | 16 | 0 | 0 | 0 |
| *Filinia* sp. | OTU6365 | 0 | 0 | 0 | 10 | 0 | 0 |
| *Filinia* sp. | OTU128 | 0 | 0 | 7 | 0 | 0 | 0 |
| *Filinia* sp. | OTU3926 | 0 | 0 | 0 | 2 | 7 | 0 |
| *Filinia* sp. | OTU5327 | 0 | 0 | 6 | 0 | 0 | 2 |
| *Filinia* sp. | OTU2598 | 0 | 0 | 7 | 1 | 0 | 0 |
| *Filinia* sp. | OTU8443 | 0 | 3 | 0 | 0 | 0 | 0 |
| *Filinia longiseta* | OTU8385 | 9 | 0 | 1 | 5 | 2 | 0 |
| *Filinia longiseta* | OTU966 | 0 | 0 | 16 | 0 | 0 | 3 |
| *Limnias melicerta* | OTU6725 | 0 | 0 | 0 | 5 | 0 | 0 |
| *Beauchampia crucigera* | OTU8201 | 0 | 0 | 0 | 0 | 15 | 0 |
| *Beauchampia crucigera* | OTU7098 | 4 | 0 | 0 | 0 | 0 | 0 |
| *Ascomorpha ovalis* | OTU700 | 47 | 18 | 6 | 21 | 0 | 18 |
| *Ascomorpha ovalis* | OTU4888 | 0 | 9 | 0 | 2 | 7 | 0 |
| *Ascomorpha ovalis* | OTU1759 | 0 | 5 | 0 | 3 | 0 | 1 |
| *Ascomorpha ovalis* | OTU5230 | 0 | 6 | 0 | 0 | 0 | 0 |
| *Hexarthra* sp. | OTU2075 | 119 | 58 | 91 | 15 | 110 | 3 |
| *Hexarthra* sp. | OTU6111 | 0 | 44 | 0 | 5 | 65 | 0 |
| *Hexarthra* sp. | OTU1717 | 0 | 5 | 0 | 0 | 3 | 0 |
| *Hexarthra* sp. | OTU2734 | 0 | 0 | 7 | 0 | 1 | 0 |
| *Pleuretra lineata* | OTU2812 | 0 | 0 | 0 | 0 | 8 | 0 |
| *Pleuretra lineata* | OTU4787 | 0 | 0 | 0 | 0 | 8 | 0 |
| *Synchaeta* sp. | OTU3113 | 0 | 16 | 2063 | 153 | 159 | 79 |
| *Synchaeta* sp. | OTU1137 | 0 | 3 | 1284 | 75 | 140 | 41 |
| *Synchaeta* sp. | OTU3283 | 0 | 0 | 1239 | 84 | 190 | 0 |
| *Synchaeta* sp. | OTU7154 | 0 | 9 | 482 | 42 | 56 | 17 |
| *Synchaeta* sp. | OTU3792 | 0 | 1 | 259 | 36 | 31 | 0 |
| *Synchaeta* sp. | OTU7058 | 35 | 11 | 72 | 36 | 94 | 46 |
| *Synchaeta* sp. | OTU1886 | 0 | 0 | 185 | 27 | 5 | 0 |
| *Synchaeta* sp. | OTU6726 | 0 | 0 | 172 | 2 | 2 | 0 |
| *Synchaeta* sp. | OTU6480 | 0 | 0 | 133 | 0 | 0 | 0 |
| *Synchaeta* sp. | OTU6165 | 0 | 0 | 106 | 13 | 9 | 0 |
| *Synchaeta* sp. | OTU4290 | 0 | 0 | 115 | 9 | 2 | 0 |
| *Synchaeta* sp. | OTU7393 | 0 | 0 | 107 | 0 | 0 | 0 |
| *Synchaeta* sp. | OTU2502 | 0 | 0 | 101 | 1 | 0 | 0 |
| *Synchaeta* sp. | OTU4038 | 0 | 0 | 98 | 0 | 1 | 0 |
| *Synchaeta* sp. | OTU5814 | 9 | 0 | 52 | 3 | 0 | 0 |
| *Synchaeta* sp. | OTU6905 | 0 | 0 | 55 | 0 | 1 | 0 |
| *Synchaeta* sp. | OTU7794 | 0 | 0 | 35 | 1 | 0 | 0 |
| *Synchaeta* sp. | OTU8056 | 3 | 3 | 2 | 3 | 17 | 12 |
| *Synchaeta* sp. | OTU7337 | 0 | 4 | 19 | 4 | 14 | 0 |
| *Synchaeta* sp. | OTU4791 | 0 | 0 | 2 | 13 | 10 | 3 |
| *Synchaeta* sp. | OTU648 | 0 | 3 | 3 | 4 | 3 | 9 |
| *Synchaeta* sp. | OTU3499 | 0 | 3 | 6 | 4 | 12 | 5 |
| *Synchaeta* sp. | OTU724 | 0 | 0 | 11 | 1 | 6 | 0 |
| *Synchaeta* sp. | OTU864 | 0 | 4 | 1 | 0 | 3 | 6 |
| *Synchaeta* sp. | OTU6854 | 0 | 0 | 9 | 0 | 0 | 0 |
| *Synchaeta* sp. | OTU3156 | 0 | 0 | 8 | 0 | 0 | 0 |
| *Synchaeta* sp. | OTU6837 | 0 | 0 | 0 | 0 | 0 | 8 |
| *Synchaeta* sp. | OTU507 | 0 | 0 | 3 | 0 | 0 | 0 |
| *Synchaeta* sp. | OTU2942 | 0 | 0 | 3 | 0 | 1 | 0 |
| *Polyarthra* sp. | OTU1858 | 0 | 3684 | 62 | 1340 | 650 | 2 |
| *Polyarthra* sp. | OTU6639 | 2 | 2251 | 54 | 960 | 374 | 16 |
| *Polyarthra* sp. | OTU8460 | 34 | 211 | 121 | 153 | 155 | 253 |
| *Polyarthra* sp. | OTU4834 | 0 | 203 | 0 | 94 | 69 | 0 |
| *Polyarthra* sp. | OTU6486 | 5 | 61 | 103 | 73 | 87 | 22 |
| *Polyarthra* sp. | OTU5269 | 0 | 9 | 199 | 4 | 0 | 80 |
| *Polyarthra* sp. | OTU778 | 11 | 25 | 59 | 43 | 5 | 12 |
| *Polyarthra* sp. | OTU934 | 0 | 33 | 0 | 77 | 11 | 0 |
| *Polyarthra* sp. | OTU5686 | 0 | 37 | 23 | 24 | 51 | 3 |
| *Polyarthra* sp. | OTU1167 | 0 | 22 | 4 | 12 | 24 | 42 |
| *Polyarthra* sp. | OTU7547 | 0 | 39 | 0 | 21 | 8 | 0 |
| *Polyarthra* sp. | OTU5845 | 0 | 52 | 0 | 9 | 4 | 0 |
| *Polyarthra* sp. | OTU4891 | 0 | 37 | 0 | 10 | 7 | 0 |
| *Polyarthra* sp. | OTU2672 | 0 | 0 | 0 | 44 | 3 | 0 |
| *Polyarthra* sp. | OTU4642 | 0 | 10 | 6 | 18 | 6 | 3 |
| *Polyarthra* sp. | OTU6740 | 7 | 2 | 2 | 4 | 11 | 15 |
| *Polyarthra* sp. | OTU4589 | 0 | 19 | 0 | 12 | 5 | 0 |
| *Polyarthra* sp. | OTU5451 | 0 | 1 | 0 | 30 | 1 | 0 |
| *Polyarthra* sp. | OTU977 | 0 | 17 | 0 | 9 | 4 | 0 |
| *Polyarthra* sp. | OTU2804 | 0 | 19 | 0 | 6 | 4 | 0 |
| *Polyarthra* sp. | OTU4387 | 0 | 17 | 0 | 6 | 1 | 0 |
| *Polyarthra* sp. | OTU1967 | 0 | 14 | 0 | 5 | 2 | 0 |
| *Polyarthra* sp. | OTU1803 | 0 | 14 | 0 | 6 | 0 | 0 |
| *Polyarthra* sp. | OTU1697 | 21 | 6 | 0 | 0 | 0 | 2 |
| *Polyarthra* sp. | OTU5503 | 0 | 11 | 0 | 5 | 2 | 0 |
| *Polyarthra* sp. | OTU872 | 0 | 4 | 0 | 0 | 0 | 0 |
| *Polyarthra* sp. | OTU5911 | 0 | 9 | 0 | 5 | 0 | 0 |
| *Polyarthra* sp. | OTU1364 | 0 | 8 | 0 | 2 | 4 | 0 |
| *Polyarthra* sp. | OTU5691 | 0 | 7 | 0 | 5 | 1 | 0 |
| *Polyarthra* sp. | OTU7466 | 0 | 9 | 0 | 3 | 0 | 0 |
| *Polyarthra* sp. | OTU1842 | 0 | 5 | 0 | 5 | 1 | 0 |
| *Polyarthra* sp. | OTU4629 | 0 | 6 | 0 | 4 | 0 | 0 |
| *Polyarthra* sp. | OTU2796 | 0 | 4 | 0 | 5 | 0 | 0 |
| *Polyarthra* sp. | OTU5306 | 0 | 6 | 0 | 5 | 0 | 0 |
| *Polyarthra* sp. | OTU6906 | 0 | 0 | 0 | 0 | 7 | 1 |
| *Polyarthra* sp. | OTU5182 | 0 | 6 | 0 | 2 | 0 | 0 |
| *Polyarthra* sp. | OTU8167 | 0 | 5 | 0 | 3 | 0 | 0 |
| *Polyarthra* sp. | OTU652 | 0 | 7 | 0 | 0 | 0 | 0 |
| *Polyarthra* sp. | OTU1289 | 0 | 6 | 0 | 1 | 0 | 0 |
| *Polyarthra* sp. | OTU7747 | 0 | 4 | 0 | 2 | 1 | 0 |
| *Polyarthra* sp. | OTU4537 | 0 | 5 | 0 | 2 | 0 | 0 |
| *Polyarthra* sp. | OTU5944 | 0 | 0 | 4 | 0 | 3 | 1 |
| *Polyarthra* sp. | OTU4610 | 0 | 3 | 0 | 1 | 2 | 0 |
| *Polyarthra* sp. | OTU5014 | 0 | 4 | 0 | 2 | 0 | 0 |
| *Polyarthra* sp. | OTU5331 | 0 | 4 | 0 | 2 | 0 | 0 |
| *Polyarthra* sp. | OTU5675 | 0 | 5 | 0 | 1 | 0 | 0 |
| *Polyarthra* sp. | OTU3788 | 0 | 3 | 0 | 2 | 0 | 0 |
| *Polyarthra* sp. | OTU7665 | 0 | 5 | 0 | 1 | 0 | 0 |
| *Polyarthra* sp. | OTU387 | 0 | 4 | 0 | 0 | 0 | 0 |
| *Polyarthra* sp. | OTU3511 | 0 | 4 | 0 | 0 | 0 | 0 |
| *Polyarthra* sp. | OTU927 | 0 | 3 | 0 | 1 | 0 | 0 |
| *Polyarthra* sp. | OTU1107 | 0 | 4 | 0 | 0 | 0 | 0 |
| *Polyarthra* sp. | OTU2604 | 0 | 3 | 0 | 2 | 0 | 0 |
| *Polyarthra* sp. | OTU2928 | 0 | 1 | 0 | 2 | 0 | 2 |
| *Polyarthra* sp. | OTU6211 | 0 | 4 | 0 | 1 | 0 | 0 |
| *Polyarthra* sp. | OTU1439 | 0 | 0 | 0 | 4 | 0 | 0 |
| *Polyarthra* sp. | OTU2132 | 0 | 3 | 0 | 1 | 0 | 0 |
| *Polyarthra* sp. | OTU4674 | 0 | 0 | 2 | 1 | 1 | 0 |
| *Polyarthra* sp. | OTU7429 | 0 | 0 | 0 | 3 | 0 | 0 |
| *Polyarthra* sp. | OTU587 | 0 | 3 | 0 | 1 | 0 | 0 |
| *Polyarthra* sp. | OTU3690 | 0 | 0 | 2 | 0 | 0 | 0 |
| *Polyarthra* sp. | OTU6516 | 0 | 3 | 0 | 0 | 0 | 0 |
| *Polyarthra* sp. | OTU2078 | 0 | 2 | 0 | 0 | 0 | 0 |
| *Polyarthra* sp. | OTU4657 | 0 | 2 | 0 | 2 | 0 | 0 |
| *Polyarthra* sp. | OTU5957 | 0 | 3 | 0 | 0 | 0 | 0 |
| *Polyarthra* sp. | OTU6244 | 0 | 1 | 0 | 2 | 0 | 0 |
| *Polyarthra* sp. | OTU6073 | 0 | 2 | 0 | 1 | 0 | 0 |
| *Polyarthra* sp. | OTU8371 | 0 | 0 | 0 | 3 | 0 | 0 |
| *Trichocerca* sp. | OTU6440 | 0 | 133 | 21 | 135 | 349 | 1 |
| *Trichocerca* sp. | OTU3465 | 60 | 56 | 352 | 48 | 152 | 0 |
| *Trichocerca* sp. | OTU2135 | 0 | 97 | 0 | 355 | 84 | 0 |
| *Trichocerca* sp. | OTU612 | 174 | 4 | 2 | 4 | 127 | 16 |
| *Trichocerca* sp. | OTU2053 | 0 | 48 | 82 | 116 | 26 | 0 |
| *Trichocerca* sp. | OTU6934 | 0 | 31 | 43 | 135 | 24 | 21 |
| *Trichocerca* sp. | OTU1599 | 63 | 33 | 79 | 17 | 17 | 2 |
| *Trichocerca* sp. | OTU3407 | 0 | 22 | 8 | 3 | 166 | 8 |
| *Trichocerca* sp. | OTU4388 | 0 | 59 | 6 | 26 | 73 | 2 |
| *Trichocerca* sp. | OTU73 | 143 | 2 | 0 | 3 | 0 | 0 |
| *Trichocerca* sp. | OTU4843 | 0 | 5 | 5 | 14 | 123 | 18 |
| *Trichocerca* sp. | OTU5866 | 0 | 28 | 1 | 9 | 68 | 0 |
| *Trichocerca* sp. | OTU3171 | 0 | 20 | 48 | 21 | 0 | 0 |
| *Trichocerca* sp. | OTU80 | 0 | 3 | 21 | 3 | 63 | 0 |
| *Trichocerca* sp. | OTU7460 | 15 | 18 | 5 | 4 | 24 | 1 |
| *Trichocerca* sp. | OTU3772 | 0 | 5 | 8 | 21 | 4 | 4 |
| *Trichocerca* sp. | OTU3177 | 0 | 15 | 0 | 21 | 4 | 0 |
| *Trichocerca* sp. | OTU1333 | 0 | 11 | 0 | 0 | 21 | 0 |
| *Trichocerca* sp. | OTU6860 | 0 | 3 | 8 | 14 | 8 | 6 |
| *Trichocerca* sp. | OTU5565 | 0 | 12 | 1 | 5 | 17 | 0 |
| *Trichocerca* sp. | OTU1081 | 0 | 0 | 0 | 0 | 40 | 0 |
| *Trichocerca* sp. | OTU2066 | 0 | 0 | 24 | 11 | 0 | 0 |
| *Trichocerca* sp. | OTU6260 | 0 | 8 | 6 | 17 | 3 | 0 |
| *Trichocerca* sp. | OTU3938 | 0 | 10 | 0 | 4 | 9 | 0 |
| *Trichocerca* sp. | OTU1742 | 4 | 8 | 1 | 3 | 3 | 1 |
| *Trichocerca* sp. | OTU3833 | 0 | 0 | 0 | 2 | 16 | 0 |
| *Trichocerca* sp. | OTU4520 | 0 | 2 | 0 | 2 | 11 | 0 |
| *Trichocerca* sp. | OTU2151 | 0 | 3 | 0 | 10 | 2 | 0 |
| *Trichocerca* sp. | OTU6450 | 0 | 0 | 7 | 0 | 9 | 0 |
| *Trichocerca* sp. | OTU8453 | 0 | 5 | 4 | 5 | 0 | 0 |
| *Trichocerca* sp. | OTU5920 | 0 | 6 | 4 | 3 | 0 | 0 |
| *Trichocerca* sp. | OTU2190 | 8 | 0 | 0 | 0 | 3 | 0 |
| *Trichocerca* sp. | OTU138 | 0 | 4 | 0 | 2 | 4 | 0 |
| *Trichocerca* sp. | OTU35 | 0 | 2 | 2 | 5 | 0 | 0 |
| *Trichocerca* sp. | OTU2122 | 0 | 0 | 0 | 0 | 9 | 0 |
| *Trichocerca* sp. | OTU6011 | 0 | 0 | 0 | 6 | 0 | 0 |
| *Trichocerca* sp. | OTU6366 | 0 | 3 | 3 | 0 | 0 | 0 |
| *Trichocerca* sp. | OTU1375 | 0 | 0 | 0 | 3 | 1 | 0 |
| *Trichocerca* sp. | OTU5543 | 0 | 3 | 0 | 3 | 0 | 0 |
| *Trichocerca* sp. | OTU5005 | 0 | 0 | 0 | 0 | 3 | 0 |
| *Trichocerca* sp. | OTU4023 | 0 | 0 | 0 | 0 | 3 | 0 |
| *Trichocerca* sp. | OTU4550 | 0 | 0 | 0 | 2 | 1 | 0 |
| *Trichocerca* sp. | OTU8423 | 0 | 0 | 0 | 1 | 2 | 0 |
| *Trichocerca* sp. | OTU6870 | 0 | 0 | 2 | 0 | 2 | 0 |
| *Trichocerca* sp. | OTU6050 | 0 | 0 | 0 | 0 | 2 | 0 |
| *Trichocerca stylata* | OTU1699 | 0 | 240 | 3 | 2419 | 142 | 3 |
| *Trichocerca stylata* | OTU6804 | 0 | 1352 | 1 | 108 | 216 | 2 |
| *Trichocerca stylata* | OTU5714 | 0 | 23 | 0 | 96 | 4 | 0 |
| *Trichotria tetractis* | OTU7510 | 0 | 26 | 0 | 4 | 0 | 0 |
| *Testudinella clypeata* | OTU7371 | 20 | 0 | 3 | 0 | 0 | 0 |
| *Testudinella clypeata* | OTU736 | 0 | 5 | 0 | 5 | 0 | 0 |
| *Testudinella patina* | OTU2376 | 0 | 0 | 0 | 7 | 0 | 0 |
| *Testudinella patina* | OTU1320 | 0 | 0 | 0 | 3 | 0 | 0 |
| *Rotaria rotatoria* | OTU5242 | 244 | 0 | 0 | 0 | 0 | 0 |
| *Rotaria rotatoria* | OTU5690 | 9 | 0 | 0 | 0 | 0 | 0 |
| *Rotaria tardigrada* | OTU7940 | 42 | 0 | 0 | 0 | 0 | 0 |
| *Rotaria neptunoida* | OTU1246 | 14 | 0 | 0 | 0 | 0 | 0 |
| Rotifera sp. | OTU5414 | 0 | 0 | 5 | 1 | 299 | 0 |
| Rotifera sp. | OTU2228 | 27 | 12 | 14 | 12 | 9 | 0 |
| Rotifera sp. | OTU6932 | 0 | 9 | 1 | 16 | 28 | 7 |
| Rotifera sp. | OTU4305 | 0 | 2 | 5 | 3 | 15 | 0 |
| Rotifera sp. | OTU2816 | 3 | 0 | 0 | 5 | 2 | 2 |
| Rotifera sp. | OTU1067 | 0 | 0 | 0 | 0 | 8 | 0 |
| Rotifera sp. | OTU3933 | 0 | 0 | 1 | 0 | 4 | 0 |
| Rotifera sp. | OTU386 | 0 | 0 | 0 | 4 | 0 | 0 |
| Rotifera sp. | OTU2526 | 0 | 0 | 0 | 0 | 3 | 0 |
| Rotifera sp. | OTU5753 | 2 | 2 | 0 | 0 | 0 | 0 |
| *Collothecaceae* sp. | OTU1527 | 0 | 0 | 0 | 0 | 0 | 2 |
| *Polyarthra dolichoptera* | OTU4649 | 0 | 0 | 72 | 0 | 0 | 0 |
| *Polyarthra dolichoptera* | OTU6706 | 0 | 0 | 36 | 0 | 4 | 0 |
| *Polyarthra dolichoptera* | OTU3037 | 0 | 3 | 2 | 0 | 34 | 2 |
| *Polyarthra dolichoptera* | OTU590 | 0 | 0 | 0 | 6 | 0 | 0 |
| *Platyias quadricornis* | OTU3955 | 0 | 0 | 15 | 0 | 0 | 0 |
| *Platyias quadricornis* | OTU7277 | 0 | 0 | 13 | 0 | 0 | 0 |
| *Platyias quadricornis* | OTU6025 | 0 | 0 | 9 | 0 | 0 | 0 |
| *Platyias quadricornis* | OTU8122 | 0 | 0 | 5 | 0 | 0 | 0 |
| *Platyias quadricornis* | OTU6649 | 0 | 0 | 4 | 0 | 0 | 0 |
| *Platyias quadricornis* | OTU6349 | 0 | 0 | 4 | 0 | 0 | 0 |
| *Synchaeta kitina* | OTU2891 | 0 | 0 | 0 | 0 | 28 | 0 |
| *Synchaeta pectinata* | OTU6169 | 0 | 0 | 0 | 0 | 10 | 0 |
| *Synchaetidae* sp. | OTU8246 | 0 | 5 | 352 | 151 | 623 | 2 |
| *Synchaetidae* sp. | OTU174 | 0 | 1 | 398 | 63 | 288 | 15 |
| *Synchaetidae* sp. | OTU4557 | 0 | 0 | 19 | 3 | 17 | 0 |
| *Synchaeta tremula* | OTU2900 | 0 | 0 | 0 | 0 | 0 | 12 |
| *Trichocercidae* sp. | OTU718 | 0 | 0 | 0 | 15 | 0 | 0 |
| *Trichocercidae* sp. | OTU1733 | 0 | 0 | 0 | 6 | 0 | 0 |
| *Trichocercidae* sp. | OTU2986 | 0 | 0 | 2 | 0 | 2 | 0 |
| *Acanthocyclops vernalis* | OTU6369 | 45 | 5 | 19 | 5 | 8 | 11 |
| *Acanthocyclops vernalis* | OTU5633 | 0 | 10 | 3 | 1 | 3 | 0 |
| *Acanthocyclops vernalis* | OTU6784 | 11 | 0 | 0 | 0 | 0 | 0 |
| *Cyclops* sp. | OTU5207 | 3267 | 647 | 500 | 52 | 15 | 509 |
| *Cyclops abyssorum* | OTU7989 | 0 | 0 | 10 | 0 | 0 | 0 |
| *Cyclops abyssorum* | OTU3235 | 0 | 3 | 2 | 0 | 0 | 0 |
| *Eucyclops serrulatus* | OTU3454 | 14 | 92 | 706 | 146 | 0 | 0 |
| *Eucyclops serrulatus* | OTU2809 | 0 | 0 | 11 | 0 | 0 | 0 |
| *Eucyclops* sp. | OTU3874 | 4 | 0 | 0 | 0 | 2 | 0 |
| *Eucyclops* sp. | OTU6677 | 0 | 3 | 0 | 0 | 4 | 0 |
| *Mesocyclops thermocyclopoides* | OTU6198 | 3 | 0 | 2 | 0 | 4 | 0 |
| *Mesocyclops thermocyclopoides* | OTU6159 | 1 | 0 | 1 | 0 | 3 | 0 |
| *Mesocyclops* sp. | OTU5213 | 10 | 395 | 1992 | 4604 | 130 | 3 |
| *Mesocyclops* sp. | OTU3451 | 10 | 33 | 1740 | 1056 | 180 | 0 |
| *Mesocyclops* sp. | OTU8474 | 1869 | 68 | 603 | 308 | 18 | 176 |
| *Mesocyclops* sp. | OTU1942 | 0 | 1000 | 93 | 153 | 21 | 212 |
| *Mesocyclops* sp. | OTU6512 | 0 | 20 | 498 | 249 | 3 | 0 |
| *Mesocyclops* sp. | OTU3592 | 2 | 8 | 244 | 93 | 25 | 0 |
| *Mesocyclops* sp. | OTU8077 | 8 | 16 | 98 | 148 | 1 | 56 |
| *Mesocyclops* sp. | OTU4994 | 5 | 9 | 68 | 106 | 3 | 0 |
| *Mesocyclops* sp. | OTU3645 | 0 | 20 | 66 | 27 | 6 | 3 |
| *Mesocyclops* sp. | OTU355 | 1 | 5 | 21 | 59 | 0 | 0 |
| *Mesocyclops* sp. | OTU6565 | 29 | 1 | 23 | 22 | 2 | 5 |
| *Mesocyclops* sp. | OTU3357 | 0 | 0 | 0 | 0 | 0 | 12 |
| *Mesocyclops* sp. | OTU343 | 0 | 31 | 10 | 20 | 4 | 0 |
| *Mesocyclops* sp. | OTU8268 | 0 | 4 | 11 | 26 | 0 | 0 |
| *Mesocyclops* sp. | OTU249 | 24 | 0 | 18 | 9 | 1 | 2 |
| *Mesocyclops* sp. | OTU7434 | 0 | 2 | 23 | 12 | 0 | 1 |
| *Mesocyclops* sp. | OTU6596 | 3 | 2 | 13 | 19 | 0 | 0 |
| *Mesocyclops* sp. | OTU5513 | 21 | 0 | 12 | 8 | 2 | 1 |
| *Mesocyclops* sp. | OTU1005 | 1 | 0 | 26 | 8 | 0 | 0 |
| *Mesocyclops* sp. | OTU865 | 0 | 0 | 0 | 5 | 7 | 36 |
| *Mesocyclops* sp. | OTU6925 | 2 | 0 | 23 | 7 | 2 | 0 |
| *Mesocyclops* sp. | OTU5632 | 0 | 0 | 24 | 5 | 3 | 0 |
| *Mesocyclops* sp. | OTU6313 | 9 | 0 | 14 | 9 | 1 | 0 |
| *Mesocyclops* sp. | OTU2402 | 6 | 0 | 17 | 3 | 7 | 0 |
| *Mesocyclops* sp. | OTU4228 | 1 | 0 | 17 | 6 | 3 | 0 |
| *Mesocyclops* sp. | OTU6622 | 0 | 0 | 7 | 11 | 1 | 0 |
| *Mesocyclops* sp. | OTU4338 | 0 | 1 | 16 | 3 | 0 | 0 |
| *Mesocyclops* sp. | OTU3060 | 3 | 1 | 10 | 7 | 0 | 0 |
| *Mesocyclops* sp. | OTU7104 | 0 | 0 | 1 | 14 | 0 | 0 |
| *Mesocyclops* sp. | OTU4463 | 0 | 0 | 13 | 2 | 0 | 0 |
| *Mesocyclops* sp. | OTU7917 | 1 | 0 | 8 | 6 | 0 | 0 |
| *Mesocyclops* sp. | OTU6105 | 0 | 6 | 2 | 6 | 0 | 0 |
| *Mesocyclops* sp. | OTU171 | 5 | 0 | 3 | 0 | 6 | 0 |
| *Mesocyclops* sp. | OTU5589 | 0 | 0 | 9 | 1 | 2 | 0 |
| *Mesocyclops* sp. | OTU8415 | 0 | 0 | 10 | 0 | 0 | 0 |
| *Mesocyclops* sp. | OTU1218 | 0 | 0 | 3 | 5 | 0 | 0 |
| *Mesocyclops* sp. | OTU2439 | 0 | 0 | 10 | 0 | 0 | 0 |
| *Mesocyclops* sp. | OTU7211 | 0 | 0 | 7 | 0 | 1 | 0 |
| *Mesocyclops* sp. | OTU7768 | 0 | 0 | 7 | 1 | 0 | 0 |
| *Mesocyclops* sp. | OTU2413 | 0 | 0 | 5 | 2 | 0 | 0 |
| *Mesocyclops* sp. | OTU7385 | 0 | 0 | 0 | 8 | 0 | 0 |
| *Mesocyclops* sp. | OTU4591 | 0 | 0 | 7 | 0 | 0 | 0 |
| *Mesocyclops* sp. | OTU2222 | 0 | 0 | 2 | 4 | 0 | 0 |
| *Mesocyclops* sp. | OTU5651 | 0 | 0 | 4 | 2 | 0 | 0 |
| *Mesocyclops* sp. | OTU3802 | 3 | 0 | 1 | 0 | 3 | 0 |
| *Mesocyclops* sp. | OTU7692 | 0 | 0 | 6 | 0 | 1 | 0 |
| *Mesocyclops* sp. | OTU1698 | 0 | 0 | 5 | 0 | 0 | 0 |
| *Mesocyclops* sp. | OTU4624 | 3 | 0 | 2 | 0 | 0 | 0 |
| *Mesocyclops* sp. | OTU6988 | 0 | 0 | 6 | 0 | 0 | 0 |
| *Mesocyclops* sp. | OTU5955 | 2 | 0 | 3 | 0 | 2 | 0 |
| *Mesocyclops* sp. | OTU1261 | 0 | 0 | 5 | 0 | 0 | 0 |
| *Mesocyclops* sp. | OTU8189 | 0 | 0 | 4 | 0 | 0 | 0 |
| *Mesocyclops* sp. | OTU357 | 0 | 0 | 6 | 0 | 0 | 0 |
| *Mesocyclops* sp. | OTU6047 | 0 | 0 | 4 | 0 | 1 | 0 |
| *Mesocyclops* sp. | OTU5487 | 2 | 0 | 4 | 0 | 0 | 0 |
| *Mesocyclops* sp. | OTU325 | 0 | 0 | 4 | 0 | 0 | 0 |
| *Mesocyclops* sp. | OTU5910 | 0 | 0 | 0 | 1 | 2 | 0 |
| *Mesocyclops* sp. | OTU3125 | 0 | 0 | 3 | 0 | 0 | 0 |
| *Macrocyclops albidus* | OTU15 | 0 | 0 | 0 | 162 | 8 | 0 |
| *Mesocyclops pehpeiensis* | OTU3167 | 0 | 0 | 0 | 0 | 29 | 0 |
| *Mesocyclops pehpeiensis* | OTU7089 | 0 | 0 | 0 | 0 | 6 | 0 |
| *Mesocyclops pehpeiensis* | OTU3214 | 0 | 2 | 0 | 3 | 0 | 0 |
| *Mesocyclops pehpeiensis* | OTU7782 | 0 | 1 | 1 | 0 | 0 | 0 |
| *Mesocyclops pehpeiensis* | OTU8228 | 0 | 4 | 1 | 0 | 0 | 0 |
| *Megacyclops viridis* | OTU4983 | 0 | 0 | 0 | 11 | 0 | 0 |
| *Thermocyclops decipiens* | OTU6641 | 5 | 2 | 0 | 2 | 0 | 0 |
| *Thermocyclops taihokuensis* | OTU4966 | 93 | 51 | 18 | 3 | 8 | 0 |
| *Thermocyclops taihokuensis* | OTU4957 | 3 | 73 | 11 | 15 | 0 | 0 |
| *Thermocyclops taihokuensis* | OTU876 | 13 | 9 | 22 | 12 | 5 | 0 |
| *Thermocyclops taihokuensis* | OTU1540 | 4 | 13 | 19 | 7 | 0 | 2 |
| *Thermocyclops taihokuensis* | OTU5297 | 2 | 1 | 0 | 0 | 1 | 0 |
| *Thermocyclops taihokuensis* | OTU6400 | 0 | 2 | 0 | 0 | 0 | 0 |
| *Thermocyclops taihokuensis* | OTU3969 | 4 | 0 | 0 | 0 | 0 | 0 |
| *Sinocalanus tenellus* | OTU6656 | 211 | 245 | 381 | 211 | 1169 | 221 |
| *Sinocalanus tenellus* | OTU6455 | 14 | 122 | 91 | 14 | 161 | 28 |
| *Sinocalanus tenellus* | OTU4974 | 24 | 18 | 29 | 5 | 54 | 19 |
| *Sinocalanus tenellus* | OTU7515 | 16 | 29 | 32 | 7 | 33 | 15 |
| *Sinocalanus tenellus* | OTU6030 | 13 | 25 | 29 | 3 | 24 | 13 |
| *Sinocalanus tenellus* | OTU984 | 4 | 19 | 22 | 4 | 21 | 11 |
| *Sinocalanus tenellus* | OTU4936 | 5 | 17 | 18 | 1 | 23 | 15 |
| *Sinocalanus tenellus* | OTU477 | 3 | 4 | 13 | 4 | 51 | 10 |
| *Sinocalanus tenellus* | OTU8218 | 6 | 21 | 12 | 0 | 30 | 7 |
| *Sinocalanus tenellus* | OTU2388 | 4 | 20 | 10 | 1 | 18 | 9 |
| *Sinocalanus tenellus* | OTU4938 | 5 | 13 | 16 | 1 | 22 | 11 |
| *Sinocalanus tenellus* | OTU7314 | 0 | 3 | 59 | 0 | 6 | 0 |
| *Sinocalanus tenellus* | OTU2371 | 6 | 12 | 9 | 0 | 15 | 8 |
| *Sinocalanus tenellus* | OTU2507 | 21 | 9 | 12 | 0 | 8 | 13 |
| *Sinocalanus tenellus* | OTU4728 | 0 | 11 | 26 | 2 | 6 | 3 |
| *Sinocalanus tenellus* | OTU861 | 1 | 7 | 11 | 0 | 13 | 6 |
| *Sinocalanus tenellus* | OTU21 | 11 | 11 | 4 | 0 | 11 | 2 |
| *Sinocalanus tenellus* | OTU6630 | 2 | 7 | 10 | 0 | 14 | 3 |
| *Sinocalanus tenellus* | OTU420 | 4 | 3 | 4 | 5 | 11 | 3 |
| *Sinocalanus tenellus* | OTU2926 | 0 | 3 | 15 | 0 | 19 | 0 |
| *Sinocalanus tenellus* | OTU8467 | 0 | 8 | 3 | 0 | 8 | 2 |
| *Sinocalanus tenellus* | OTU4429 | 8 | 5 | 6 | 1 | 0 | 8 |
| *Sinocalanus tenellus* | OTU7786 | 0 | 5 | 1 | 0 | 8 | 0 |
| *Sinocalanus tenellus* | OTU2594 | 0 | 5 | 6 | 0 | 12 | 1 |
| *Sinocalanus tenellus* | OTU4714 | 3 | 6 | 0 | 0 | 4 | 7 |
| *Sinocalanus tenellus* | OTU5502 | 3 | 3 | 2 | 1 | 7 | 4 |
| *Sinocalanus tenellus* | OTU6838 | 0 | 3 | 1 | 0 | 14 | 0 |
| *Sinocalanus tenellus* | OTU505 | 9 | 2 | 3 | 0 | 0 | 5 |
| *Sinocalanus tenellus* | OTU3746 | 11 | 5 | 0 | 0 | 0 | 0 |
| *Sinocalanus tenellus* | OTU5472 | 10 | 0 | 1 | 3 | 0 | 0 |
| *Sinocalanus tenellus* | OTU4736 | 0 | 3 | 3 | 0 | 5 | 2 |
| *Sinocalanus tenellus* | OTU4264 | 2 | 5 | 3 | 0 | 1 | 0 |
| *Sinocalanus tenellus* | OTU1182 | 0 | 0 | 4 | 1 | 5 | 0 |
| *Sinocalanus tenellus* | OTU5808 | 0 | 3 | 3 | 0 | 2 | 1 |
| *Sinocalanus tenellus* | OTU1531 | 0 | 1 | 2 | 0 | 2 | 0 |
| *Sinocalanus tenellus* | OTU3133 | 2 | 0 | 1 | 0 | 2 | 1 |
| *Sinocalanus tenellus* | OTU4617 | 0 | 4 | 0 | 0 | 4 | 0 |
| *Sinocalanus tenellus* | OTU890 | 1 | 4 | 1 | 0 | 1 | 1 |
| *Sinocalanus tenellus* | OTU1977 | 0 | 9 | 2 | 0 | 0 | 0 |
| *Sinocalanus tenellus* | OTU8013 | 1 | 0 | 0 | 1 | 4 | 2 |
| *Sinocalanus tenellus* | OTU2567 | 0 | 0 | 1 | 0 | 7 | 0 |
| *Sinocalanus tenellus* | OTU6449 | 7 | 2 | 0 | 1 | 0 | 0 |
| *Sinocalanus tenellus* | OTU6698 | 0 | 3 | 1 | 1 | 4 | 0 |
| *Sinocalanus tenellus* | OTU1931 | 2 | 0 | 1 | 0 | 1 | 0 |
| *Sinocalanus tenellus* | OTU3641 | 0 | 9 | 0 | 0 | 0 | 0 |
| *Sinocalanus tenellus* | OTU4220 | 0 | 0 | 0 | 5 | 0 | 0 |
| *Sinocalanus tenellus* | OTU6294 | 0 | 0 | 0 | 1 | 1 | 2 |
| *Sinocalanus tenellus* | OTU393 | 1 | 2 | 1 | 0 | 3 | 0 |
| *Sinocalanus tenellus* | OTU378 | 6 | 1 | 0 | 0 | 0 | 0 |
| *Sinocalanus tenellus* | OTU7050 | 0 | 3 | 0 | 0 | 1 | 0 |
| *Sinocalanus tenellus* | OTU2639 | 0 | 0 | 2 | 0 | 2 | 1 |
| *Sinocalanus tenellus* | OTU8269 | 2 | 0 | 2 | 0 | 0 | 0 |
| *Sinocalanus tenellus* | OTU5528 | 0 | 1 | 1 | 0 | 0 | 0 |
| *Calanoides natalis* | OTU3940 | 0 | 0 | 0 | 0 | 6 | 0 |
| *Heliodiaptomus kikuchii* | OTU4600 | 0 | 1 | 0 | 2 | 0 | 11 |
| *Neodiaptomus schmackeri* | OTU3751 | 0 | 46 | 0 | 7 | 0 | 0 |
| *Neodiaptomus schmackeri* | OTU8255 | 0 | 3 | 0 | 2 | 0 | 0 |
| *Neodiaptomus schmackeri* | OTU1755 | 5 | 1 | 0 | 0 | 0 | 0 |
| *Prionodiaptomus colombiensis* | OTU2417 | 9565 | 0 | 0 | 0 | 0 | 0 |
| *Skistodiaptomus pallidus* | OTU2562 | 88 | 0 | 0 | 0 | 0 | 0 |
| *Skistodiaptomus pallidus* | OTU2273 | 7 | 0 | 0 | 0 | 0 | 0 |
| Cyclopidae sp. | OTU8000 | 0 | 0 | 0 | 18 | 1 | 0 |
| Cyclopoida sp. | OTU7575 | 300 | 640 | 5885 | 5215 | 2520 | 23 |
| Cyclopoida sp. | OTU411 | 129 | 403 | 1369 | 1179 | 23 | 0 |
| Cyclopoida sp. | OTU2403 | 94 | 250 | 159 | 136 | 217 | 12 |
| Cyclopoida sp. | OTU1476 | 127 | 23 | 3 | 3 | 0 | 416 |
| Cyclopoida sp. | OTU4821 | 0 | 39 | 79 | 56 | 6 | 2 |
| Cyclopoida sp. | OTU150 | 3 | 12 | 69 | 32 | 10 | 0 |
| Cyclopoida sp. | OTU2790 | 2 | 8 | 43 | 19 | 0 | 0 |
| Cyclopoida sp. | OTU8451 | 0 | 0 | 27 | 4 | 15 | 0 |
| Cyclopoida sp. | OTU1921 | 0 | 0 | 17 | 4 | 8 | 0 |
| Cyclopoida sp. | OTU5260 | 0 | 1 | 19 | 7 | 2 | 0 |
| Cyclopoida sp. | OTU8118 | 0 | 1 | 8 | 15 | 0 | 0 |
| Cyclopoida sp. | OTU6729 | 4 | 3 | 6 | 0 | 6 | 0 |
| Cyclopoida sp. | OTU5758 | 0 | 0 | 12 | 0 | 4 | 0 |
| Cyclopoida sp. | OTU1016 | 0 | 1 | 8 | 4 | 0 | 0 |
| Cyclopoida sp. | OTU4101 | 2 | 0 | 10 | 0 | 2 | 0 |
| Cyclopoida sp. | OTU5863 | 1 | 2 | 3 | 0 | 4 | 0 |
| Cyclopoida sp. | OTU6525 | 0 | 0 | 3 | 1 | 3 | 0 |
| Cyclopoida sp. | OTU3712 | 0 | 0 | 4 | 1 | 2 | 0 |
| Cyclopoida sp. | OTU6292 | 0 | 0 | 5 | 0 | 0 | 0 |
| Cyclopoida sp. | OTU5761 | 0 | 0 | 4 | 0 | 0 | 0 |
| Cyclopoida sp. | OTU6659 | 0 | 0 | 3 | 0 | 1 | 0 |
| Cyclopoida sp. | OTU6039 | 0 | 0 | 4 | 0 | 0 | 0 |
| Cyclopoida sp. | OTU5363 | 0 | 0 | 5 | 0 | 0 | 0 |
| Cyclopoida sp. | OTU8035 | 0 | 0 | 1 | 0 | 1 | 0 |
| Cyclopoida sp. | OTU8216 | 0 | 0 | 0 | 4 | 0 | 0 |
| Cyclopoida sp. | OTU1228 | 0 | 0 | 2 | 1 | 0 | 0 |
| Cyclopoida sp. | OTU1885 | 0 | 0 | 0 | 2 | 1 | 0 |
| Cyclopoida sp. | OTU4711 | 2 | 0 | 2 | 0 | 0 | 0 |
| Cyclopoida sp. | OTU3393 | 0 | 0 | 2 | 0 | 2 | 0 |
| Cyclopoida sp. | OTU8124 | 0 | 0 | 4 | 0 | 0 | 0 |
| *Bosmina fatalis* | OTU2171 | 6018 | 17974 | 2016 | 102 | 6712 | 6380 |
| *Bosmina fatalis* | OTU3210 | 776 | 2059 | 153 | 3 | 454 | 1394 |
| *Bosmina fatalis* | OTU2418 | 2286 | 581 | 100 | 1 | 253 | 175 |
| *Bosmina fatalis* | OTU4932 | 89 | 133 | 0 | 0 | 4 | 5 |
| *Bosmina fatalis* | OTU3444 | 1 | 43 | 14 | 0 | 31 | 22 |
| *Bosmina fatalis* | OTU7848 | 108 | 12 | 0 | 0 | 3 | 2 |
| *Bosmina fatalis* | OTU6604 | 54 | 4 | 0 | 0 | 0 | 0 |
| *Bosmina fatalis* | OTU86 | 36 | 8 | 0 | 0 | 0 | 7 |
| *Bosmina fatalis* | OTU2644 | 6 | 22 | 0 | 0 | 2 | 17 |
| *Bosmina fatalis* | OTU8213 | 0 | 10 | 2 | 0 | 7 | 5 |
| *Bosmina fatalis* | OTU92 | 24 | 1 | 0 | 0 | 0 | 0 |
| *Bosmina fatalis* | OTU1971 | 0 | 9 | 2 | 0 | 4 | 4 |
| *Bosmina fatalis* | OTU3080 | 0 | 6 | 0 | 0 | 7 | 1 |
| *Bosmina fatalis* | OTU4174 | 8 | 3 | 0 | 0 | 0 | 0 |
| *Bosmina fatalis* | OTU814 | 0 | 5 | 0 | 0 | 3 | 2 |
| *Bosmina fatalis* | OTU2014 | 1 | 6 | 0 | 0 | 0 | 3 |
| *Bosmina fatalis* | OTU5496 | 6 | 1 | 0 | 0 | 0 | 0 |
| *Bosmina fatalis* | OTU7739 | 6 | 3 | 0 | 0 | 0 | 0 |
| *Bosmina fatalis* | OTU3092 | 7 | 0 | 0 | 0 | 1 | 0 |
| *Bosmina fatalis* | OTU3399 | 7 | 1 | 0 | 0 | 0 | 0 |
| *Bosmina fatalis* | OTU4063 | 0 | 2 | 0 | 0 | 4 | 0 |
| *Bosmina fatalis* | OTU6317 | 7 | 0 | 0 | 0 | 0 | 0 |
| *Bosmina fatalis* | OTU3247 | 0 | 7 | 1 | 0 | 0 | 0 |
| *Bosmina fatalis* | OTU2530 | 0 | 3 | 0 | 0 | 4 | 0 |
| *Bosmina fatalis* | OTU3295 | 1 | 7 | 0 | 0 | 0 | 0 |
| *Bosmina fatalis* | OTU439 | 2 | 3 | 0 | 0 | 0 | 1 |
| *Bosmina fatalis* | OTU8055 | 0 | 1 | 0 | 0 | 1 | 1 |
| *Bosmina fatalis* | OTU6017 | 1 | 6 | 1 | 0 | 0 | 0 |
| *Bosmina fatalis* | OTU3428 | 0 | 4 | 0 | 0 | 0 | 0 |
| *Bosmina fatalis* | OTU2027 | 3 | 1 | 0 | 0 | 0 | 0 |
| *Bosmina fatalis* | OTU3375 | 3 | 0 | 0 | 0 | 0 | 0 |
| *Bosmina fatalis* | OTU6246 | 0 | 7 | 0 | 0 | 0 | 0 |
| *Bosmina fatalis* | OTU3211 | 0 | 3 | 0 | 0 | 0 | 0 |
| *Bosmina fatalis* | OTU3730 | 0 | 2 | 0 | 0 | 0 | 0 |
| *Bosmina fatalis* | OTU4702 | 0 | 2 | 0 | 0 | 0 | 1 |
| *Bosmina fatalis* | OTU6252 | 2 | 0 | 0 | 0 | 0 | 0 |
| *Bosmina longirostris* | OTU6900 | 0 | 61 | 0 | 0 | 0 | 17 |
| *Bosmina longirostris* | OTU7631 | 0 | 23 | 0 | 0 | 0 | 6 |
| *Bosmina longirostris* | OTU8033 | 3 | 3 | 0 | 0 | 0 | 0 |
| Bosminidae sp. | OTU651 | 139 | 0 | 0 | 0 | 0 | 0 |
| Bosminidae sp. | OTU6936 | 9 | 33 | 6 | 0 | 8 | 23 |
| Bosminidae sp. | OTU957 | 2 | 7 | 0 | 0 | 0 | 7 |
| Bosminidae sp. | OTU2086 | 14 | 0 | 0 | 0 | 0 | 0 |
| Bosminidae sp. | OTU6610 | 13 | 0 | 0 | 0 | 0 | 0 |
| Bosminidae sp. | OTU644 | 0 | 3 | 1 | 0 | 3 | 2 |
| Bosminidae sp. | OTU4427 | 4 | 0 | 0 | 0 | 0 | 0 |
| Chydoridae sp. | OTU472 | 0 | 47 | 3 | 0 | 0 | 26 |
| Chydoridae sp. | OTU3664 | 0 | 4 | 26 | 0 | 0 | 0 |
| Chydoridae sp. | OTU6556 | 0 | 0 | 23 | 0 | 0 | 0 |
| Chydoridae sp. | OTU8529 | 0 | 0 | 9 | 0 | 2 | 0 |
| *Chydorus sphaericus* | OTU5016 | 0 | 0 | 0 | 0 | 11 | 0 |
| *Alona* sp. | OTU7504 | 0 | 0 | 4 | 0 | 0 | 0 |
| *Ceriodaphnia cornuta* | OTU4756 | 0 | 226 | 0 | 0 | 3 | 0 |
| *Ceriodaphnia* sp. | OTU6524 | 0 | 239 | 0 | 0 | 0 | 0 |
| *Ceriodaphnia* sp. | OTU8144 | 0 | 0 | 0 | 0 | 18 | 0 |
| *Ceriodaphnia* sp. | OTU5601 | 0 | 0 | 0 | 0 | 5 | 0 |
| *Leptodora richardi* | OTU5173 | 0 | 1 | 0 | 223 | 0 | 0 |
| *Leptodora richardi* | OTU3743 | 0 | 0 | 0 | 5 | 0 | 0 |
| *Macrothrix* sp. | OTU4462 | 798 | 13 | 8 | 19 | 57 | 8 |
| *Macrothrix* sp. | OTU6091 | 10 | 5 | 188 | 8 | 45 | 62 |
| *Macrothrix* sp. | OTU5113 | 22 | 4 | 0 | 0 | 30 | 17 |
| *Macrothrix* sp. | OTU5498 | 27 | 10 | 4 | 5 | 34 | 4 |
| *Macrothrix* sp. | OTU7729 | 19 | 5 | 2 | 3 | 8 | 9 |
| *Macrothrix* sp. | OTU1934 | 1 | 0 | 4 | 0 | 4 | 10 |
| *Macrothrix* sp. | OTU2019 | 0 | 0 | 0 | 0 | 0 | 3 |
| *Macrothrix* sp. | OTU8530 | 0 | 0 | 3 | 0 | 0 | 0 |
| *Macrothrix spinosa* | OTU7217 | 0 | 8 | 0 | 22 | 0 | 0 |
| *Moina* sp. | OTU2882 | 9 | 285 | 740 | 1276 | 37 | 0 |
| *Moina* sp. | OTU2717 | 0 | 0 | 5 | 0 | 25 | 0 |
| *Latonopsis australis* | OTU1812 | 36 | 0 | 0 | 0 | 0 | 0 |
| *Daphnia galeata* | OTU7353 | 7 | 2 | 0 | 15 | 0 | 0 |
| *Daphnia galeata* | OTU7881 | 0 | 0 | 0 | 6 | 0 | 0 |
| *Daphnia galeata* | OTU2283 | 0 | 0 | 0 | 5 | 0 | 0 |
| *Daphnia galeata* | OTU6378 | 0 | 0 | 0 | 3 | 0 | 0 |
| *Diaphanosoma dubium* | OTU5070 | 0 | 0 | 1331 | 16 | 0 | 0 |
| *Diaphanosoma dubium* | OTU1578 | 0 | 0 | 355 | 17 | 0 | 0 |
| *Diaphanosoma dubium* | OTU5658 | 0 | 0 | 186 | 2 | 10 | 0 |
| *Diaphanosoma dubium* | OTU5594 | 0 | 1 | 103 | 69 | 0 | 0 |
| *Diaphanosoma dubium* | OTU701 | 0 | 69 | 81 | 40 | 0 | 0 |
| *Diaphanosoma dubium* | OTU1135 | 0 | 0 | 59 | 0 | 0 | 0 |
| *Diaphanosoma dubium* | OTU6225 | 0 | 0 | 26 | 9 | 0 | 0 |
| *Diaphanosoma dubium* | OTU1082 | 0 | 0 | 33 | 0 | 0 | 0 |
| *Diaphanosoma dubium* | OTU7084 | 0 | 0 | 13 | 0 | 0 | 0 |
| *Diaphanosoma dubium* | OTU2225 | 0 | 0 | 13 | 0 | 0 | 0 |
| *Diaphanosoma dubium* | OTU3701 | 0 | 0 | 4 | 0 | 0 | 0 |
| *Diaphanosoma dubium* | OTU7835 | 0 | 0 | 3 | 1 | 0 | 0 |
| *Diaphanosoma dubium* | OTU5365 | 0 | 0 | 3 | 0 | 0 | 0 |
| *Diaphanosoma orghidani* | OTU8108 | 0 | 0 | 4 | 0 | 3 | 0 |
| *Diaphanosoma* sp. | OTU6579 | 0 | 0 | 12 | 0 | 0 | 0 |
